# ﻿Additions to the coriaceous families Peniophoraceae and Stereaceae (Russulales): Six novel wood-inhabiting taxa in the genera *Conferticium*, *Gloeocystidiellum*, and *Peniophora* from southwest China

**DOI:** 10.3897/mycokeys.115.147044

**Published:** 2025-03-21

**Authors:** Lu Wang, Yonggao Zhu, Siyuan He, Sana Jabeen, Changlin Zhao

**Affiliations:** 1 The Key Laboratory of Forest Resources Conservation and Utilization in the Southwest Mountains of China Ministry of Education, Key Laboratory of National Forestry and Grassland Administration on Biodiversity Conservation in Southwest China, Yunnan Provincial Key Laboratory for Conservation and Utilization of In-forest Resource, Southwest Forestry University, Kunming 650224, China Southwest Forestry University Kunming China; 2 College of Forestry, Southwest Forestry University, Kunming 650224, China University of Education Lahore Pakistan; 3 Department of Botany, Division of Science and Technology, University of Education, Lahore, Punjab, Pakistan Southwest Forestry University Kunming China; 4 Yunnan Academy of Biodiversity, Southwest Forestry University, Kunming 650224, China University of Education Lahore Pakistan

**Keywords:** Biodiversity, corticioid fungi, molecular systematics, taxonomy, Yunnan Province

## Abstract

Russulales comprises a highly diverse group of species with respect to basidiomata morphology and hymenophore configuration, in which this order is highly heterogeneous, that can be classified as resupinate, effused-reflexed, discoid, clavarioid, pileate, or stipitate, and with varied hymenophores such as smooth, hydnoid, poroid, and lamellate in different russuloid species. Species in Russulales have been considered to have significant economic value. Six new wood-inhabiting fungi belonging to the genera *Conferticium*, *Gloeocystidiellum*, and *Peniophora* of two families, Peniophoraceae and Stereaceae (Russulales), were found in southwest China. Sequences of ITS+nLSU loci of six new taxa were generated, and phylogenetic analyses were performed with the maximum likelihood, maximum parsimony, and Bayesian inference methods with an emphasis on the phylogeny of wood-inhabiting smooth species in this order. The combined ITS+nLSU loci analysis showed that the six new species grouped within the order Russulales, in which *Conferticiumtuberculatum*, *Gloeocystidiellumcremeum*, and *G.fissuratum* grouped into the family Stereaceae, and *Peniophoraalbohymenia*, *P.hengduanensis*, and *P.punctata* grouped into the family Peniophoraceae. The morphology and multi-gene phylogenetic analyses confirmed the novelty and placement of the six new taxa. Descriptions, illustrations, and phylogenetic analysis results of the new taxa are provided.

## ﻿Introduction

The order Russulales Kreisel ex P.M. Kirk, P.F. Cannon & J.C. David is a highly diverse group of the class Agaricomycetes, which includes around 4,436 described species in 98 genera and 11 families (Hyde et al. 2017; Caboň et al. 2019; Vidal et al. 2019; He et al. 2024). Russulales contains not only the lamellate species like *Russula* Pers. and *Lactarius* Pers. (Herrera et al. 2018; Caboň et al. 2019) but also the poroid, hydnoid, and corticioid representatives like *Asterostroma* Massee, *Hericium* Pers., *Heterobasidion* Bref., and *Peniophora* Cooke (Miller et al. 2006; Chen et al. 2016a; Wu et al. 2020; Yuan et al. 2021; Zhou et al. 2024). In the past few decades, a lot of the new taxa of Russulales have been gathered through both morphological characteristics and DNA sequence phylogenetic analyses (Zhou and Dai 2013; Chen et al. 2015, 2016b; Wu et al. 2020; Zou et al. 2022; Bhunjun et al. 2024; Deng et al. 2024b; Dong et al. 2024; Zhou et al. 2024). Resupinate basidiomata are common in the families Echinodontiaceae, Peniophoraceae, Stereaceae, Terrestriporiaceae, and Xenasmataceae (He et al. 2024; Liu et al. 2024).

The genus *Conferticium* Hallenb. 1980 (Stereaceae, Russulales), typified by *C. insidiosum* (Bourdot & Galzin) Hallenb. (Bernicchia and Gorjón 2010), is characterized by the resupinate basidiomata with membranaceous to ceraceous, smooth to tuberculate hymenophore, a monomitic hyphal system with simple-septate, and numerous cylindrical, sinuous gloeocystidia (Bernicchia and Gorjón 2010). Based on the MycoBank database (http://www.mycobank.org, accessed on 26 February 2025) and the Index Fungorum (http://www.indexfungorum.org, accessed on 26 February 2025), *Conferticium* has registered six specific and infraspecific names; however, only five species are widely recognized (Larsson and Larsson 2003; Bernicchia and Gorjón 2010).

Donk (1931) described *Gloeocystidiellum* (Stereaceae, Russulales), typified by *G.porosum* (Berk. and M.A. Curtis) Donk, as characterized by their resupinate basidiomata with membranaceous to ceraceous, smooth, rarely grandinioid or odontioid hymenophore, a monomitic hyphal system with simple-septate or clamped generative hyphae, and the gloeocystidia numerous, verrucose or aculeate basidiospores (Wu 1996; Bernicchia and Gorjón 2010; Zhao and Zhao 2023). Based on the MycoBank database (http://www.mycobank.org, accessed on 26 February 2025) and the Index Fungorum (http://www.indexfungorum.org, accessed on 26 February 2025), *Gloeocystidiellum* has registered 86 specific and infraspecific names, and the actual number of species is 37 (Zhao and Zhao 2023).

The genus *Peniophora* Cooke (Peniophoraceae, Russulales) was introduced in 1879, typified by *P.quercina* Pers. ex Fr., and it is characterized by the resupinate basidiomata with a smooth hymenophore, a monomitic hyphal system, thin- to thick-walled simple-septate or clamped generative hyphae, dendrohyphidia, lamprocystidia, and gloeocystidia present or absent, and thin-walled, smooth, ellipsoid, cylindrical to allantoid basidiospores negative in Melzer’s reagents (Bernicchia and Gorjón 2010; Zou et al. 2022; Xu et al. 2023). Species of the genus prefer to grow on small branches of trees, especially dead but still attached ones in exposed and dry environments (Xu et al. 2023). Based on the MycoBank database (http://www.mycobank.org, accessed on 26 February 2025) and the Index Fungorum (http://www.indexfungorum.org, accessed on 26 February 2025), *Peniophora* was registered with 657 specific and infraspecific names, and the actual number of the species is 211, in which most species have been moved to other genera, and the morphological circumscription of *Peniophora* has been narrowed (Xu et al. 2023). Currently, they occur mainly in the tropical and subtropical areas of the world (Zou et al. 2022; Xu et al. 2023).

Recently, the analysis of DNA sequences has emerged as a common method for deducing fungal phylogenies and enhancing higher classification frameworks through the integration of genetic traits (Wijayawardene et al. 2022, 2024; Dong et al. 2024; He et al. 2022, 2024; Zhang et al. 2023b; Zhao et al. 2023; Zhou et al. 2023; 2025). According to recent research on molecular systematics and divergence times, Basidiomycota is classified into 127 families belonging to 47 orders under 14 classes (He et al. 2024). The family Stereaceae included nineteen genera: *Acanthobasidium*, *Acanthofungus*, *Acanthophysellum*, *Aleurobotrys*, *Aleurodiscus*, *Amylohyphus*, *Amylosporomyces*, *Coniophorafomes*, *Dextrinocystidium*, *Gloeocystidiellum*, *Gloeocystidiopsis*, *Gloeomyces*, *Gloeosoma*, *Megalocystidium*, *Neoaleurodiscus*, *Scotoderma*, *Stereodiscus*, *Stereum*, and *Xylobolus* (Larsson 2007; Vu et al. 2019; He et al. 2024). According to recent research on molecular systematics, the genus *Conferticium* has been reported to have one new taxon, *C. fissuratum* Xin Yang & C.L. Zhao, which is from Yunnan Province, China (Bernicchia and Gorjón 2010; Shen et al. 2024). In the previous study, the phylogenetic relationships among russuloid basidiomycetes were investigated using sequence data from the nuclear 5.8S, ITS2, and large-subunit rDNA genes and elucidated evolutionary relationships within some species of the genus *Gloeocystidiellum* (Larsson and Larsson 2003). The high phylogenetic diversity among corticioid homobasidiomycetes indicated that the species *G.subasperisporum* (Litsch.) J. Erikss. & Ryvarden grouped into the russuloid clade and grouped with the close species *Gloeodontiadiscolor* (Berk. & M.A. Curtis) Boidin (Larsson et al. 2004). Several morphological and molecular studies as well as cultural studies and intercompatibility tests have analyzed species delimitation in the *Gloeocystidiellumporosum-clavuligerum* group, which revealed that the clade corresponded with two well-distinguished species, *G.kenyense* and *G.clavuligerum* (Telleria et al. 2012). The molecular research carried out on the genus *Gloeocystidiellum*, in which the species *G.granulatum* (Sheng H. Wu) E. Larss. & K.H. Larss and *G.permixtum* (Boidin, Lanq. & Gilles) E. Larss. & K.H. Larss are proposed as new combinations (Larsson et al. 2020). Recently, molecular studies involving *Gloeocystidiellum* based on single-locus or multi-locus datasets have introduced two new taxa, viz. *G.lojanense* A. Jaram., D. Cruz & Decock, and *G.yunnanense* Y.L. Zhao & C.L. Zhao (Jaramillo-Riofrío et al. 2023; Zhao and Zhao 2023).

The genus *Peniophora* Cooke is a large genus of corticioid fungi, which is a cosmopolitan genus with a wide distribution from boreal to tropical areas, causing a white rot on both angiosperms and gymnosperms (Yurchenko 2010; Zou et al. 2022; Xu et al. 2023). Many new lineages and taxa were found and described, and some morphologically dissimilar taxa were proved to be closely related in the phylogeny of *Peniophora* in recent years (Harrington et al. 2021; Lambevska-Hristova 2022; Zou et al. 2022; Xu et al. 2023). The ITS and nLSU sequences of *Peniophora* species, including some from type specimens, were released in GenBank in recent studies and thus made it possible to deeply study the phylogeny of this group (Vu et al. 2019; Zou et al. 2022). The researchers performed the most comprehensive phylogenetic studies using ITS+nLSU datasets, including most of the *Peniophora* species described worldwide (Zou et al. 2022; Xu et al. 2023). Most clades in *Peniophora* are far too resolved, at least considering only ITS or nLSU rDNA regions, and probably a genome full of phylogenetic reconstruction is needed to establish with certitude groups or patterns in the evolution of the different species (Xu et al. 2023; Dong et al. 2024).

Many wood-inhabiting specimens were collected during investigations on wood-inhabiting fungi in the Yunnan-Guizhou Plateau, China. To clarify the placement and relationships of these specimens, we carried out a phylogenetic and taxonomic study based on the ITS+nLSU sequences. These specimens were assigned to the genera *Conferticium*, *Gloeocystidiellum*, and *Peniophora* of the order Russulales. Therefore, six new species, *Conferticiumtuberculatum*, *Gloeocystidiellumcremeum*, *G.fissuratum*, *Peniophoraalbohymenia*, *P.hengduanensis*, and *P.punctata*, are proposed with descriptions and illustrations based on the morphological characteristics and phylogenetic analyses.

## ﻿Materials and methods

### ﻿Sample collection and herbarium specimen preparation

Fresh basidiomata of the wood-inhabiting fungi growing on angiosperm branches were collected from the Zhaotong and Diqing of Yunnan Province, China. The samples were photographed *in situ*, and fresh macroscopic details and collection information (Rathnayaka et al. 2024) were recorded. Photographs were taken by a Jianeng 80D camera (Tokyo, Japan). All photos were stacked and merged using Helicon Focus Pro 7.7.5 software. Specimens were dried in an electric food dehydrator at 40 °C, then sealed and stored in an envelope bag and deposited in the herbarium of the Southwest Forestry University (SWFC), Kunming, Yunnan Province, China.

### ﻿Morphology

Macromorphological descriptions are based on field notes and photos captured in the field and laboratory and follow the color terminology of Petersen (1996). Micromorphological data were obtained from the dried specimens following observation under a light microscope (Zhao et al. 2023a; Dong et al. 2024). The following abbreviations were used: KOH = 5% potassium hydroxide water solution, CB+ = cyanophilous, CB = cotton clue, CB– = acyanophilous, IKI = Melzer’s reagent, IKI+ = amyloid, IKI– = both inamyloid and indextrinoid, L = means spore length (arithmetic average for all spores), W = means spore width (arithmetic average for all spores), Q = variation in the L/W ratios between the specimens studied, and n = a/b (number of spores (a) measured from a given number (b) of specimens).

### ﻿DNA extraction, amplification, and sequencing

The CTAB rapid plant genome extraction kit-DN14 (Aidlab Biotechnologies Co., Ltd., Beijing, China) was used to obtain genomic DNA from dried specimens, according to the previous study (Zhao and Wu 2017). The ITS region was amplified with the primer pair ITS5 and ITS4 (White et al. 1990). The nuclear nLSU region was amplified with primer pair LR0R and LR7 (Vilgalys and Hester 1990). The PCR procedure for ITS was as follows: initial denaturation at 95 °C for 3 min, followed by 35 cycles at 94 °C for 40 s, 54 °C for 45 s, and 72 °C for 1 min; and a final extension at 72 °C for 10 min. The PCR procedure for nLSU was as follows: initial denaturation at 94 °C for 1 min, followed by 35 cycles at 94 °C for 30 s, 50 °C for 1 min, and 72 °C for 1.5 min, and a final extension at 72 °C for 10 min. The PCR products were purified and sequenced at Kunming Tsingke Biological Technology Limited Company, Kunming, Yunnan Province, P.R. China. All newly generated sequences were deposited in GenBank (Table 1).

**Table 1.** Names, voucher numbers, references, and corresponding GenBank accession numbers of the taxa used in the phylogenetic analyses.

Taxa	Voucher	Locality	GenBank accession		Reference
			ITS	nLSU	
*Acanthobasidiumbambusicola*	He2357	China	KU559343	KU574833	Dai and He 2016
*Acanthobasidiumpenicillatum*	HHB13223	USA	—	KU574816	Maekawa et al. 2023
*Acanthofungusrimosus*	Wu 9601-1	China	MF043521	AY039333	Maekawa et al. 2023
*Acanthophysellumcerussatum*	He 20120920—3	China	KU559339	KU574830	Maekawa et al. 2023
*Acanthophysiumbisporum*	T614	USA	—	AY039327	Maekawa et al. 2023
*Acanthophysiumlividocaeruleum*	FP-100292	USA	—	AY039319	Maekawa et al. 2023
*Aleurobotrysbotryosus*	He2712	China	KX306877	KY450788	Maekawa et al. 2023
*Aleurobotrysbotryosus*	Wu 9302-61	China	—	AY039331	Maekawa et al. 2023
*Aleurodiscusbambusinus*	He4261	China	KY706207	KY706219	Yan et al. 2018
*Aleurodiscuscanadensis*	Wu1207-90	China	KY706203	KY706225	Yan et al. 2018
*Aleurodiscusmirabilis*	Dai 13281	China	KU559350	KU574839	Yan et al. 2018
*Asterostromalaxum*	EL33-99	Estonia	AF506410	AF506410	Larsson and Larsson 2003
*Asterostromamuscicola*	KHL9537	Puerto Rico	AF506409	AF506409	Larsson and Larsson 2003
*Asterostromarhizomorpharum*	CLZhao 31212	China	OR672732	OR879302	Zhou et al. 2024
*Asterostromayunnanense*	CLZhao 22781	China	OR048809	OR506285	Deng et al. 2024a
*Baltazariaoctopodites*	FLOR 56449	Brazil	MH260025	MH260047	Leal-Dutra et al. 2018
*Conferticiumheimii*	CBS321.66	African	MH858805	MH858805	Tian et al. 2018
*Conferticiumochraceum*	CLZhao 21515	China	ON211619	—	Present study
*Conferticiumochraceum*	G07_P24A	Switzerland	KT943933	—	Stroheker et al. 2018
*Conferticiumravum*	CBS:125849	Estonia	MH863805	MH875269	Vu et al. 2019
*Conferticiumravum*	NH13291	USA	AF506382	AF506382	Larsson and Larsson 2003
*Conferticiumtuberculatum*	CLZhao 29376*	China	PQ166602	PQ295861	Present study
*Conferticiumtuberculatum*	CLZhao 29390	China	PQ166603	PQ295862	Present study
*Dichostereumeffuscatum*	GG930915	France	AF506390	AF506390	Larsson and Larsson 2003
*Dichostereumpallescens*	NH7046/673	Canada	AF506392	AF506392	Larsson and Larsson 2003
*Entomocorticiumcobbii*	B720	USA	MT741707	MT741692	Harrington et al. 2021
*Entomocorticiumwhitneyi*	B1069	USA	MT741713	MT741698	Harrington et al. 2021
*Gloeocystidiellumaspellum*	He4262	China	—	KY860460	Jaramillo-Riofrío et al. 2023
*Gloeocystidiellumaspellum*	LIN 625	China	AF506432	AF506432	Yan et al. 2018
*Gloeocystidiellumbisporum*	CBS/961.96	Sweden	AY048875	AY048875	Jaramillo-Riofrío et al. 2023
*Gloeocystidiellumbisporum*	KHL11135	Norway	AY048877	AY048877	Larsson and Larsson 2003
*Gloeocystidiellumclavuligerum*	GB/NH11185	Spain	AF310088	AF310088	Jaramillo-Riofrío et al. 2023
*Gloeocystidiellumclavuligerum*	NH13159/2731	Russia	AF310083	AF310083	Larsson and Larsson 2003
*Gloeocystidiellumcompactum*	Wu880615-21	China	AF506434	AF506434	Maekawa et al. 2023
*Gloeocystidiellumcremeum*	CLZhao 29477*	China	PQ287846	PQ295863	Present study
*Gloeocystidiellumcremeum*	CLZhao 33623	China	PQ287847	PQ295864	Present study
*Gloeocystidiellumcremeum*	CLZhao 33690	China	PQ287848	—	Present study
*Gloeocystidiellumfissuratum*	CLZhao 32247	China	PQ287849	PQ295865	Present study
*Gloeocystidiellumfissuratum*	CLZhao 32303	China	PQ287850	PQ295866	Present study
*Gloeocystidiellumfissuratum*	CLZhao 32498*	China	PQ287851	—	Present study
*Gloeocystidiellumformosanum*	Wu9404-19	China	AF506439	AF506439	Maekawa et al. 2023
*Gloeocystidiellumheimii*	LY/CBS321.66	African	AF506381	AF506381	Jaramillo-Riofrío et al. 2023
*Gloeocystidiellumkenyense*	TFC/15278	Portugal	FR878082	—	Jaramillo-Riofrío et al. 2023
*Gloeocystidiellumkenyense*	TFC/15309	Portugal	FR878083	—	Jaramillo-Riofrío et al. 2023
*Gloeocystidiellumlojanense*	HUTPL(F)/2181	Ecuador	OP377059	OP377059	Jaramillo-Riofrío et al. 2023
*Gloeocystidiellumlojanense*	HUTPL(F)/550	Ecuador	OP377083	OP377083	Jaramillo-Riofrío et al. 2023
*Gloeocystidiellumluridum*	HK9808	Germany	AF506421	AF506421	Maekawa et al. 2023
*Gloeocystidiellumporosum*	CBS/51085	Netherlands	AF310097	AF310097	Jaramillo-Riofrío et al. 2023
*Gloeocystidiellumporosum*	NH 10434	Denmark	AF310094	AF310094	Larsson and Hallenberg 2001
*Gloeocystidiellumpurpureum*	Wu9310-45	China	AF441338	AF441338	Larsson and Hallenberg 2001
*Gloeocystidiellumrajchenbergii*	GB/NH16348	Chile	JQ734555	—	Jaramillo-Riofrío et al. 2023
*Gloeocystidiellumrajchenbergii*	GB/NH16358	Chile	JQ734554	—	Jaramillo-Riofrío et al. 2023
*Gloeocystidiellumtriste*	KHL10334	Sweden	AF506442	AF506442	Maekawa et al. 2023
*Gloeocystidiellumyunnanense*	CLZhao 7165	China	MZ710569	MZ710571	Zhao and Zhao 2023
*Gloeocystidiellumyunnanense*	CLZhao 7202	China	MZ710570	MZ710572	Zhao and Zhao 2023
*Gloeocystidiopsisflammea*	CBS:324.66	African	AF506437	AF506437	Larsson and Larsson 2003
*Gloeocystidiopsisheimii*	CBS:321.66	Sweden	AF506381	AF506381	Larsson and Larsson 2003
*Gloiothelelactescens*	EL8-98	Sweden	AF506453	AF506453	Larsson and Larsson 2003
*Gloiothelelamellosa*	KHL11031	Venezuela	AF506454	AF506454	Larsson and Larsson 2003
*Lachnocladiumschweinfurthianum*	KM 49740	Cameroon	MH260033	MH260051	Leal-Dutra et al. 2018
*Megalocystidiumchelidonium*	LodgeSJ110.1	USA	AF506441	AF506441	Larsson and Larsson 2003
*Megalocystidiumdiffissum*	V.Spirin4244	Sweden	MT477147	MT477147	Maekawa et al. 2023
*Megalocystidiumleucoxanthum*	HK9808	Sweden	AF506420	AF506420	Maekawa et al. 2023
*Metulodontianivea*	NH13108	Russia	AF506423	AF506423	Larsson and Larsson 2003
*Neoaleurodiscusfujii*	He2921	China	KU559357	KU574845	Dai et al. 2017
*Neoaleurodiscusfujii*	Wu0807-41	Japan	—	FJ799924	Dai et al. 2017
*Parapteruliciumsubarbusculum*	FLOR 56456	Brazil	MH260026	MH260048	Leal-Dutra et al. 2018
*Peniophoraalbobadia*	CBS:329.66	France	MH858809	MH870448	Vu et al. 2019
*Peniophoraalbobadia*	He2159	USA	MK588755	MK588795	Xu et al. 2023
*Peniophoraalbohymenia*	CLZhao 23473*	China	PQ066419	PQ295867	Present study
*Peniophoraaurantiaca*	CBS:396.50	France	MH856678	MH868195	Vu et al. 2019
*Peniophoraaurantiaca*	UBCF:19732	Canada	HQ604854	HQ604854	Xu et al. 2023
*Peniophorabicornis*	He3609	China	MK588763	MK588803	Xu et al. 2023
*Peniophorabicornis*	He4767	China	MK588764	MK588804	Xu et al. 2023
*Peniophoraborbonica*	He4597	China	MK588766	MK588806	Xu et al. 2023
*Peniophoraborbonica*	He4606	China	MK588765	MK588805	Xu et al. 2023
*Peniophoracinerea*	CLZhao 23390	China	PQ166604	PQ295868	Present study
*Peniophoracinerea*	He3725	China	MK588769	MK588809	Xu et al. 2023
*Peniophoracrassitunicata*	CLZhao 29461	China	PQ166605	PQ295869	Present study
*Peniophoracrassitunicata*	He3814	China	MK588770	MK588810	Xu et al. 2023
*Peniophoracremicolor*	He5380	China	MK588791	MK588831	Xu et al. 2023
*Peniophoraduplex*	CBS:286.58	Canada	MH857787	MH869321	Vu et al. 2019
*Peniophoraduplex*	TPDuB1022	USA	AF119519	—	Harrington and Hsaiu 2003
*Peniophoraerikssonii*	CBS:287.58	France	MH857788	MH869322	Vu et al. 2019
*Peniophoraerikssonii*	Cui 11871	China	MK588771	MK588811	Xu et al. 2023
*Peniophoraexima*	T-523	USA	MK588772	MK588812	Xu et al. 2023
*Peniophorafasticata*	CBS:942.96	Ethiopia	MH862624	—	Vu et al. 2019
*Peniophorafissilis*	CBS:681.91	Reunion	MH862298	MH873975	Vu et al. 2019
*Peniophorafissilis*	CBS:684.91	Mascarene Islands	MH862299	MH873976	Vu et al. 2019
*Peniophoragilbertsonii*	CBS:357.95	USA	MH862528	MH874164	Vu et al. 2019
*Peniophoragilbertsonii*	CBS:360.95	USA	MH862530	MH874165	Vu et al. 2019
*Peniophoraguadelupensis*	CBS:715.91	Guadeloupe	MH862304	MH873977	Vu et al. 2019
*Peniophorahalimi*	CBS:863.84	France	MH861844	MH873532	Vu et al. 2019
*Peniophorahalimi*	CBS:864.84	France	MH861845	MH873533	Vu et al. 2019
*Peniophorahengduanensis*	CLZhao 34697*	China	PQ066422	PQ295870	Present study
*Peniophoraincarnata*	CBS:398.50	France	MH856680	MH868197	Vu et al. 2019
*Peniophoraincarnata*	CBS:399.50	France	MH856681	MH868198	Vu et al. 2019
*Peniophorajunipericola*	CBS:349.54	Sweden	MH857354	—	Vu et al. 2019
*Peniophorajunipericola*	He2462	China	MK588773	MK588813	Xu et al. 2023
*Peniophorakuehneri*	CBS:719.91	Mascarene Islands	MH862307	MH873980	Vu et al. 2019
*Peniophorakuehneri*	He4745	China	MK588757	MK588797	Xu et al. 2023
*Peniophorakuehneroides*	CBS:731.91	Mascarene Islands	MH862317	MH873989	Vu et al. 2019
*Peniophorakuehneroides*	CBS:732.91	Mascarene Islands	MH862318	MH873990	Vu et al. 2019
*Peniophoralaete*	CBS:256.56	France	MH857617	MH869165	Vu et al. 2019
*Peniophoralassa*	He3052	China	MK588758	MK588798	Xu et al. 2023
*Peniophoralassa*	SP6129	Russia	KJ509191	—	Spirin and Kout 2015
*Peniophoralaxitexta*	BAFC 3309	Argentina	FJ882040	—	Robles et al. 2011
*Peniophoralaxitexta*	LGMF1159	Argentina	JX559580	—	Xu et al. 2023
*Peniophoralilacea*	CBS:337.66	Armenia	MH858813	MH870452	Vu et al. 2019
*Peniophoralimitata*	olrim 963	Lithuania	AY787678	—	Lygis et al. 2005
*Peniophoralycii*	Boid-437	France	MK588774	MK588814	Xu et al. 2023
*Peniophoralycii*	CBS:264.56	France	MH857624	MH869169	Vu et al. 2019
*Peniophoramajor*	He5528	China	MK588792	MK588832	Xu et al. 2023
*Peniophoramalaiensis*	CBS:679.91	Singapore	MH862297	MH873974	Vu et al. 2019
*Peniophoramalaiensis*	CLZhao 23595	China	PQ166607	PQ295871	Present study
*Peniophoramanshurica*	He2956	China	MK588776	MK588816	Xu et al. 2023
*Peniophoramanshurica*	He3729	China	MK588777	MK588817	Xu et al. 2023
*Peniophorameridionalis*	CBS:289.58	France	MH857789	MH869323	Vu et al. 2019
*Peniophoramolesta*	CBS:676.91	Gabon	MH862294	MH873973	Vu et al. 2019
*Peniophoramolesta*	CBS:677.91	Gabon	MH862295	—	Vu et al. 2019
*Peniophoramonticola*	CBS:649.91	Reunion	MH862289	MH873970	Vu et al. 2019
*Peniophoranuda*	CLZhao 23406	China	PQ166608	PQ295872	Present study
*Peniophoranuda*	He5280	China	MK588778	MK588818	Xu et al. 2023
*Peniophoraovalispora*	CBS:653.91	Mascarene Islands	MH862290	MH873971	Vu et al. 2019
*Peniophoraparvocystidiata*	CBS:716.91	Guadeloupe	MH862305	MH873978	Vu et al. 2019
*Peniophorapiceae*	209	Russia	JX507718	—	Grum-Grzhimaylo et al. 2016
*Peniophorapiceae*	olrim10	Sweden	AY781264	—	Vasiliauskas et al. 2005
*Peniophorapilatiana*	CBS:265.56	France	MH857625	MH869170	Vu et al. 2019
*Peniophorapilatiana*	CBS -A1/A2	China	MK588780	MK588820	Xu et al. 2023
*Peniophorapini*	CBS:274.56	France	MH857632	MH869177	Vu et al. 2019
*Peniophorapini*	Hjm 18143	Sweden	EU118651	EU118651	Larsson 2007
*Peniophorapithya*	CBS:277.56	France	MH857635	MH869180	Vu et al. 2019
*Peniophorapithya*	He3107	China	MK588781	MK588821	Xu et al. 2023
*Peniophorapolygonia*	CBS:404.50	France	MH856684	MH868201	Vu et al. 2019
*Peniophorapolygonia*	He4651	China	MK588782	MK588822	Xu et al. 2023
*Peniophoraproxima*	CBS:405.50	France	MH856685	MH868202	Vu et al. 2019
*Peniophoraproxima*	He5498	China	MK588783	MK588823	Xu et al. 2023
*Peniophorapseudopini*	DAOM-30124-Sp	Canada	MK588784	MK588824	Xu et al. 2023
*Peniophorapseudopini*	TPPpB1007	USA	AF119514	—	Leal-Dutra et al. 2018
*Peniophorapunctata*	CLZhao 33769*	China	PQ066418	—	Present study
*Peniophoraquercina*	CBS:408.50	France	MH856688	MH868205	Vu et al. 2019
*Peniophoraquercina*	CBS:407.50	France	MH856687	MH868204	Vu et al. 2019
*Peniophorareidii*	CBS:397.83	France	MH861616	MH873334	Vu et al. 2019
*Peniophorarhoica*	CBS:943.96	Ethiopia	MH862625	MH874246	Vu et al. 2019
*Peniophoraroseoalba*	CLZhao 31523	China	PQ166609	PQ295873	Present study
*Peniophoraroseoalba*	CLZhao 3513	China	ON786559	OP380690	Zou et al. 2022
*Peniophorarufa*	CBS:351.59	Canada	MH857891	MH869432	Vu et al. 2019
*Peniophorarufa*	He2788	China	MK588786	MK588826	Xu et al. 2023
*Peniophorarufomarginata*	CBS:281.56	France	MH857639	MH869183	Vu et al. 2019
*Peniophorarufomarginata*	CBS:282.56	France	MH857640	MH869184	Vu et al. 2019
*Peniophoraseptentrionalis*	CBS:294.58	Canada	MH857791	MH869325	Vu et al. 2019
*Peniophorashenghuae*	CLZhao 23654	China	PQ066420	—	Present study
*Peniophorashenghuae*	CLZhao 35044	China	PQ066421	—	Present study
*Peniophorashenghuae*	He3507	China	MK588788	MK588828	Xu et al. 2023
*Peniophorashenghuae*	He5447	China	MK588790	MK588830	Xu et al. 2023
*Peniophorasimulans*	CBS:874.84	France	MH861849	MH873537	Vu et al. 2019
*Peniophorasimulans*	CBS:875.84	France	MH861850	MH873538	Vu et al. 2019
*Peniophorasphaerocystidiata*	HHB-8827-Sp	USA	MK588787	MK588827	Xu et al. 2023
*Peniophorasubsalmonea*	CBS:696.91	Mascarene Islands	MH862302	—	Vu et al. 2019
*Peniophorasubsalmonea*	CBS:697.91	Mascarene Islands	MH862303	—	Vu et al. 2019
*Peniophorataiwanensis*	Wu9206-28	China	MK588793	MK588833	Xu et al. 2023
*Peniophorataiwanensis*	Wu9209-14	China	MK588794	MK588834	Xu et al. 2023
*Peniophoratamaricicola*	CBS:438.62	Morocco	MH858203	MH869802	Vu et al. 2019
*Peniophoratamaricicola*	CBS:439.62	Morocco	MH858204	MH869803	Vu et al. 2019
*Peniophoratrigonosperma*	CBS:402.83	France	MH861618	MH873335	Vu et al. 2019
*Peniophoratrigonosperma*	He3602	China	MK588762	MK588802	Xu et al. 2023
*Peniophoratristicula*	CBS:210.63	Pakistan	MH858266	—	Vu et al. 2019
*Peniophoratristicula*	He4775	China	MH669235	MH669239	Liu and He 2018
*Peniophoraversicolor*	CBS:358.61	Morocco	MH858082	MH869651	Vu et al. 2019
*Peniophoraversiformis*	CBS:358.54	France	MH857360	MH868902	Vu et al. 2019
*Peniophoraversiformis*	He3029	China	MK588756	MK588796	Xu et al. 2023
*Peniophoravietnamensis*	He5242	Vietnam	MK588761	MK588801	Xu et al. 2023
*Peniophoravietnamensis*	He5252	Vietnam	MK588761	MK588801	Xu et al. 2023
*Peniophoraviolaceolivida*	CBS:348.52	France	MH857077	MH868613	Vu et al. 2019
*Peniophorayunnanensis*	CLZhao 7347	China	OP380616	—	Zou et al. 2022
*Peniophorayunnanensis*	CLZhao 3978	China	OP380617	OP380689	Zou et al. 2022
*Scytinostromaacystidiatum*	CLZhao 32022	China	PQ166610	PQ295874	Present study
*Scytinostromaacystidiatum*	Dai 24608	China	OQ689127	OQ629351	Zhang et al. 2023
*Scytinostromabambusinum*	CLZhao 32789	China	PQ166599	PQ295875	Present study
*Scytinostromabambusinum*	JXH 643	China	OR510627	PP660872	Ji et al. 2024
*Scytinostromamacrospermum*	Dai 24606	China	OQ689126	OQ629350	Wang et al. 2020
*Scytinostromaportentosum*	EL11-99	Sweden	AF506470	AF506470	Larsson and Larsson 2003
*Stereodiscuslimonisporus*	CBS:125846	New Zealand	—	MH875266	Maekawa et al. 2023
*Stereumcomplicatum*	He2234	China	KU559368	KU574828	Maekawa et al. 2023
*Stereumhirsutum*	Wu1109—127	China	LC430906	LC430909	Maekawa et al. 2023
*Stereumsanguinolentum*	He2111	China	KU559367	KU574827	Maekawa et al. 2023
*Varariafissurata*	CLZhao 8171	China	OQ025219	OR539503	Deng et al. 2024b
*Varariainvestiens*	TAA164122	Norway	AF506484	AF506484	Larsson and Larsson 2003
*Varariatropica*	CBS:704.81	France	MH861447	MH873189	Vu et al. 2019
*Varariayaoshanensis*	CLZhao 20693	China	PP091665	PP091684	Deng et al. 2024b
*Vesiculomycescitrinus*	EL53-97	Sweden	AF506486	AF506486	Deng et al. 2024b
*Xylobolusfrustulatus*	He2231	USA	KU881905	KU574825	Maekawa et al. 2023
*Xylobolussubpileatus*	FP-106735	USA	—	AY039309	Maekawa et al. 2023

* indicates type materials; — indicates sequence unavailability.

### ﻿Phylogenetic analyses

**Figure 1.**
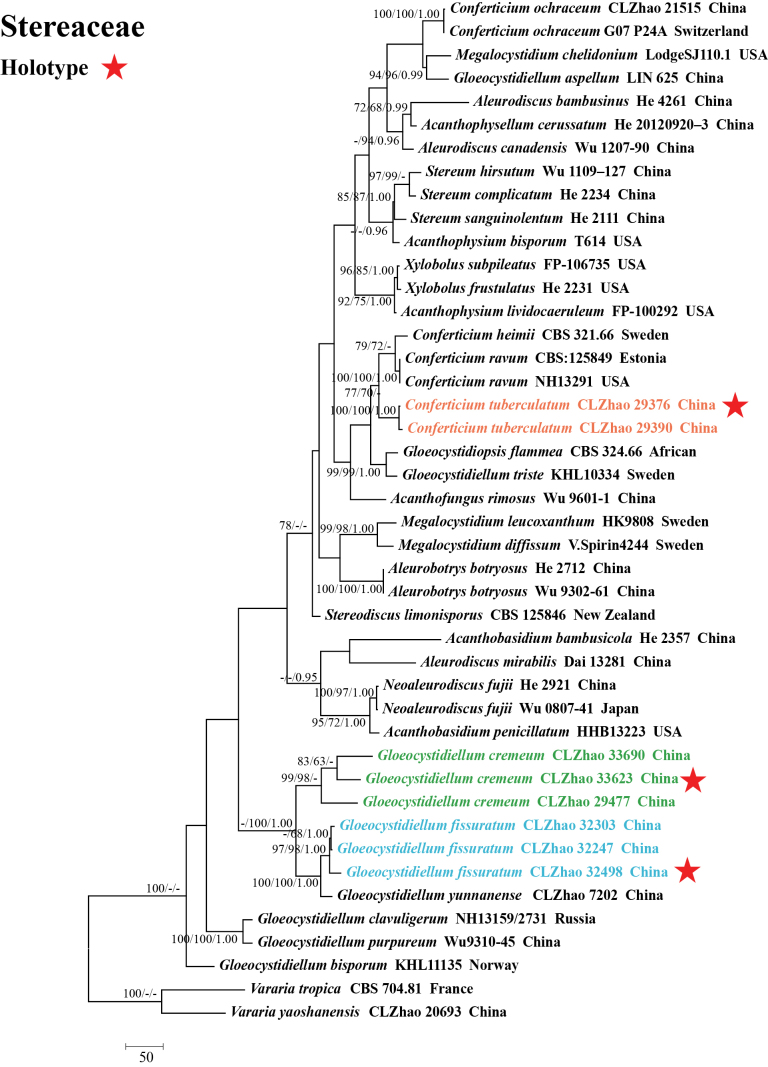
Maximum parsimony strict consensus tree illustrating the phylogeny of *Conferticium* and *Gloeocystidiellum* and related genera in the family Stereaceae, based on ITS+nLSU sequences; branches are labeled with maximum likelihood bootstrap value ≥ 70%, parsimony bootstrap value ≥ 50%, and Bayesian posterior probabilities ≥ 0.95.

**Figure 2.**
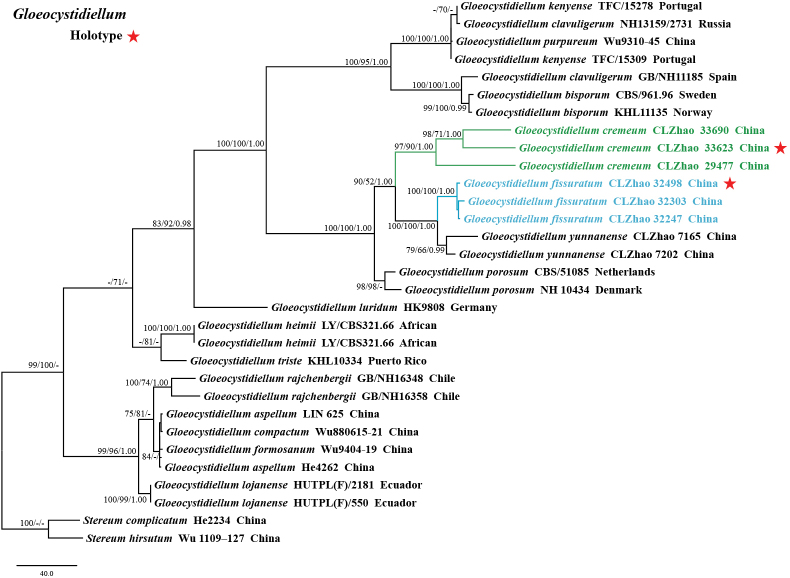
Maximum parsimony strict consensus tree illustrating the phylogeny of two new species and related species in the genus *Gloeocystidiellum*, based on ITS+nLSU sequences; branches are labeled with maximum likelihood bootstrap value ≥ 70%, parsimony bootstrap value ≥ 50%, and Bayesian posterior probabilities ≥ 0.95.

**Figure 3.**
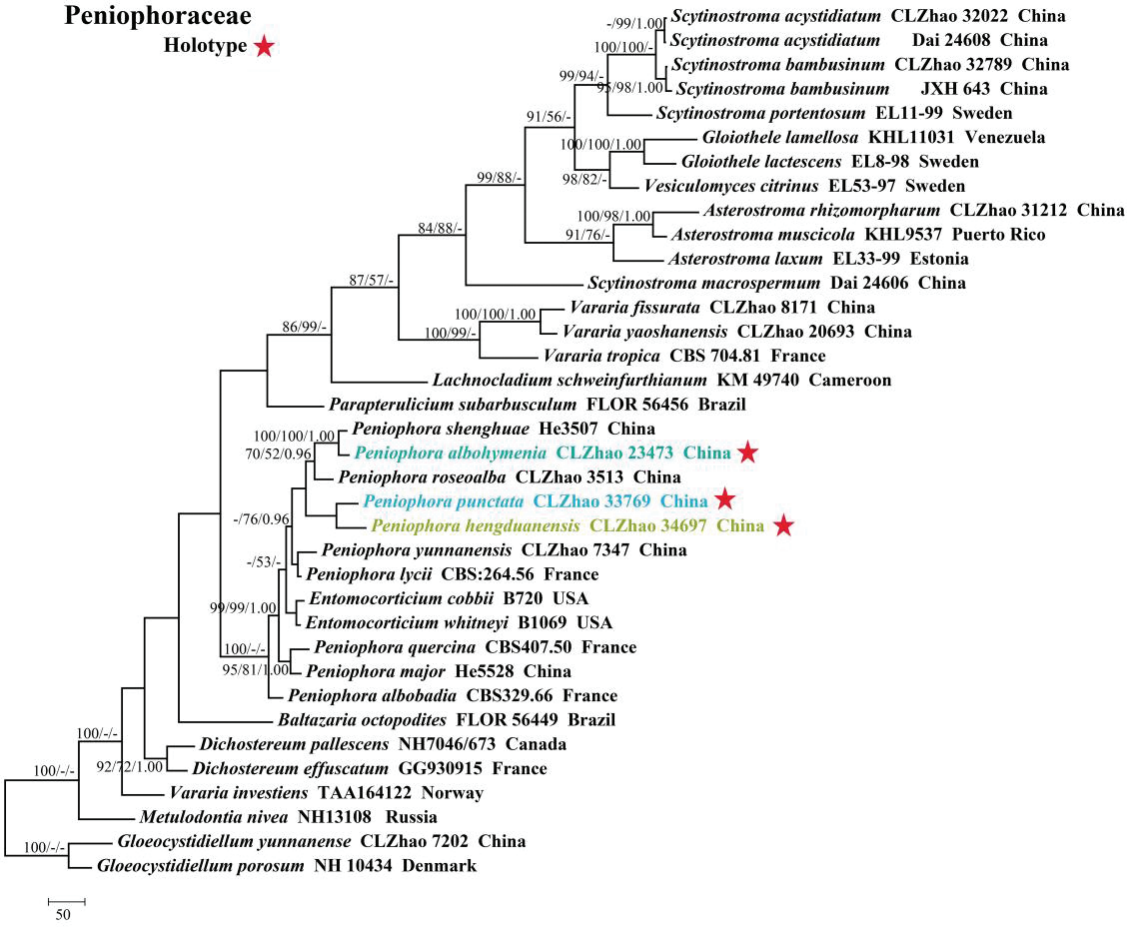
Maximum parsimony strict consensus tree illustrating the phylogeny of *Peniophora* and related genera in the family Peniophoraceae, based on ITS+nLSU sequences; branches are labeled with maximum likelihood bootstrap value ≥ 70%, parsimony bootstrap value ≥ 50%, and Bayesian posterior probabilities ≥ 0.95.

**Figure 4.**
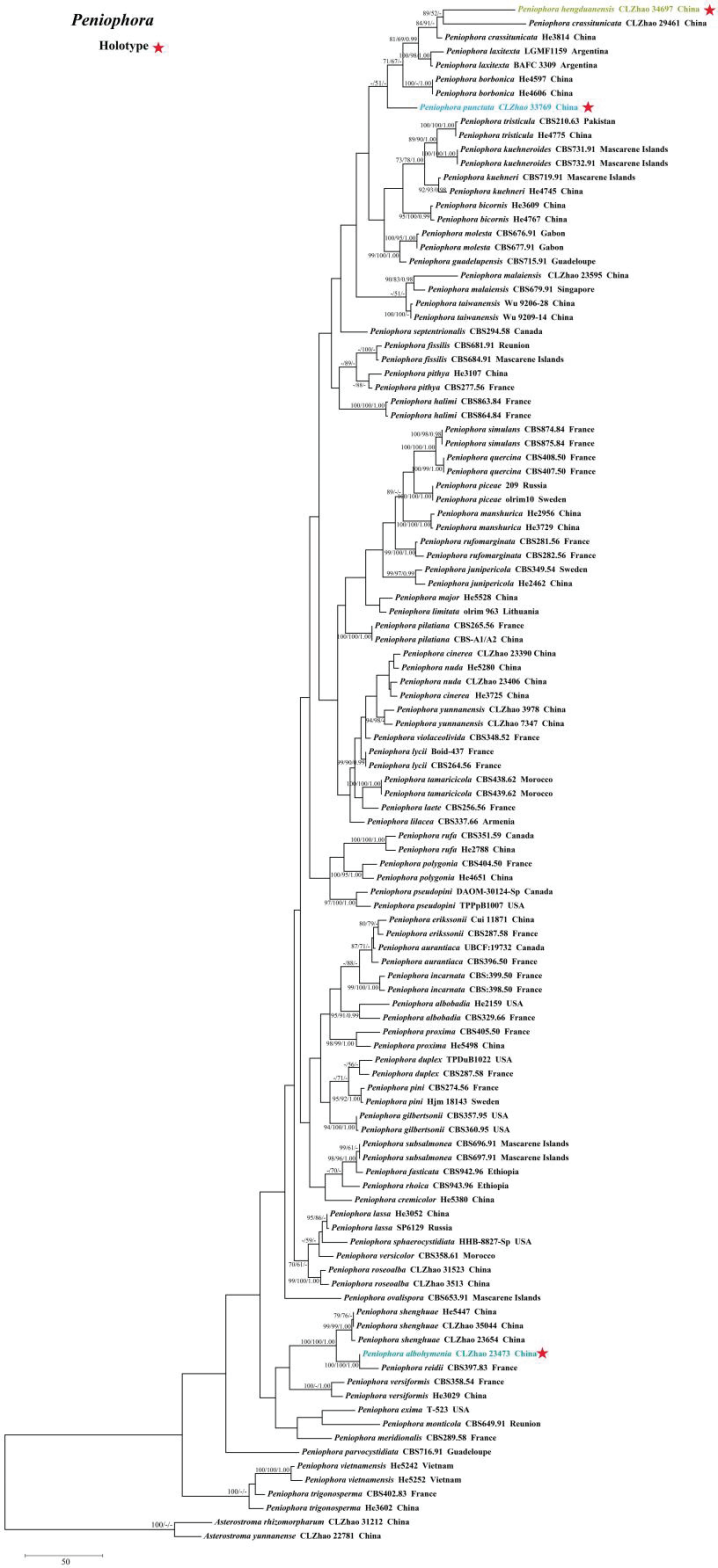
Maximum parsimony strict consensus tree illustrating the phylogeny of three new species and related species in the genus *Peniophora*, based on ITS+nLSU sequences; branches are labeled with maximum likelihood bootstrap value ≥ 70%, parsimony bootstrap value ≥ 50%, and Bayesian posterior probabilities ≥ 0.95.

Sequences were aligned using MAFFT version 7, adjusting the direction of nucleotide sequences according to the first sequence and selecting the G-INS-i iterative refinement method (Katoh et al. 2019). The alignment was adjusted manually using AliView version 1.27 (Larsson 2014). A dataset of concatenated ITS and nLSU sequences was used to determine the phylogenetic position of the six new species. (1) *Varariatropica* A.L. Welden and *V.yaoshanensis* Y.L. Deng & C.L. Zhao were assigned as outgroups to root trees in the ITS+nLSU analysis (Fig. 1) (Deng et al. 2024b); (2) *Stereumcomplicatum* (Fr.) Fr. and *S.hirsutum* (Willd.) Pers. were assigned as outgroups to root trees following the ITS+nLSU analysis (Fig. 2) (Maekawa et al. 2023); (3) *Gloeocystidiellumyunnanense* Y.L. Zhao & C.L. Zhao and *G.porosum* Berk. & M.A. Curtis) Donk were assigned as outgroups to root trees following the ITS+nLSU analysis (Fig. 3) (Zhao and Zhao 2023); (4) *Asterostromarhizomorpharum* H.M. Zhou & C.L. Zhao and *A.yunnanense* Y.L. Deng & C.L. Zhao were assigned as outgroups to root trees following the ITS+nLSU analysis (Fig. 4) (Zhou et al. 2024).

Maximum parsimony (MP), maximum likelihood (ML), and Bayesian inference (BI) analyses were applied to the combined three datasets following the methods outlined in a previous study (Zhao and Wu 2017), and the tree construction procedure was performed in PAUP* version 4.0b10 (Swofford 2002). All characters were equally weighted, and gaps were treated as missing data. Trees were inferred using the heuristic search option with TBR branch swapping and 1,000 random sequence additions. Max-trees were set to 5000, branches of zero length were collapsed, and all parsimonious trees were saved. Clade robustness was assessed using bootstrap (BT) analysis with 1,000 replicates (Felsenstein 1985). Descriptive tree statistics—tree length (TL), consistency index (CI), retention index (RI), rescaled consistency index (RC), and homoplasy index (HI)—were calculated for each maximum parsimonious tree generated. Additionally, the multiple sequence alignment was also analyzed using maximum likelihood (ML) in RAxML-HPC2 through the Cipres Science Gateway (Miller et al. 2012). Branch support (BS) for ML analysis was determined by 1000 bootstrap replicates. jModelTest v2 (Darriba et al. 2012) was used to determine the best-fit evolutionary model for each data set for Bayesian inference (BI), which was performed using MrBayes 3.2.7a (Ronquist et al. 2012). The first one-fourth of all generations was discarded as burn-in. The majority rule consensus tree of all remaining trees was calculated. Branches were considered as significantly supported if they received a maximum likelihood bootstrap value (BS) ≥ 70%, a maximum parsimony bootstrap value (BT) ≥ 50%, or Bayesian posterior probabilities (BPP) ≥ 0.95.

## ﻿Result

### ﻿The phylogeny of Stereaceae

The dataset based on ITS+nLSU (Fig. 1) comprises sequences from 44 fungal specimens representing 35 species from GenBank. The dataset had an aligned length of 2,136 characters, of which 1,324 characters are constant, 250 are variable and parsimony-uninformative, and 562 are parsimony-informative. Maximum parsimony analysis yielded five equally parsimonious trees (TL = 2,504, CI = 0.5112, HI = 0.4888, RI = 0.6001, RC = 0.3068). The best model for the ITS+nLSU dataset estimated and applied in the Bayesian analysis was GTR+I+G. The phylogenetic tree (Fig. 1) inferred from ITS+nLSU sequences revealed that *Conferticiumtuberculatum*, *Gloeocystidiellumcremeum*, and *G.fissuratum* grouped into the family Stereaceae.

### ﻿The phylogeny of *Gloeocystidiellum*

The dataset based on ITS+nLSU (Fig. 2) comprises sequences from 31 fungal specimens representing 18 species from GenBank. The dataset had an aligned length of 2,154 characters, of which 1,605 characters are constant, 131 are variable and parsimony-uninformative, and 418 are parsimony-informative. Maximum parsimony analysis yielded five equally parsimonious trees (TL = 1,039, CI = 0.7372, HI = 0.2628, RI = 0.8640, RC = 0.6370). The best model for the ITS+nLSU dataset estimated and applied in the Bayesian analysis was GTR+I+G. The phylogenetic tree (Fig. 2) inferred from ITS+nLSU sequences revealed that *Gloeocystidiellumcremeum* and *G.fissuratum* grouped into the genus *Gloeocystidiellum*.

### ﻿The phylogeny of Peniophoraceae

The dataset based on ITS+nLSU (Fig. 3) comprises sequences from 36 fungal specimens representing 34 species from GenBank. The dataset had an aligned length of 2,375 characters, of which 1,345 characters are constant, 304 are variable and parsimony-uninformative, and 726 are parsimony-informative. Maximum parsimony analysis yielded five equally parsimonious trees (TL = 3,317, CI = 0.5246, HI = 0.4754, RI = 0.6349, RC = 0.3330). The best model for the ITS+nLSU dataset estimated and applied in the Bayesian analysis was GTR+I+G. The phylogenetic tree (Fig. 3) inferred from ITS+nLSU sequences revealed that *Peniophoraalbohymenia*, *P.hengduanensis*, and *P.punctata* grouped into the family Peniophoraceae.

### ﻿The phylogeny of *Peniophora*

The dataset based on ITS+nLSU (Fig. 4) comprises sequences from 110 fungal specimens representing 66 species from GenBank. The dataset had an aligned length of 2,006 characters, of which 1,389 characters are constant, 194 are variable and parsimony-uninformative, and 423 are parsimony-informative. Maximum parsimony analysis yielded five equally parsimonious trees (TL = 2,470, CI = 0.3441, HI = 0.6559, RI = 0.6067, RC = 0.2088). The best model for the ITS+nLSU dataset estimated and applied in the Bayesian analysis was SYM+I+G. The phylogenetic tree (Fig. 4) inferred from ITS+nLSU sequences revealed that *Peniophoraalbohymenia*, *P.hengduanensis*, and *P.punctata* grouped into the genus *Peniophora*.

### ﻿Taxonomy

#### ﻿ Conferticium tuberculatum

Taxon classificationFungiRussulalesStereaceae

L. Wang & C.L. Zhao sp. nov.

10861E23-D144-587A-B039-D249409D0FB9

855872

Figs 5 , 6

##### Typification.

China. • Yunnan Province: Zhaotong, Daguan County, Huanglianhe Scenic Spot, GPS coordinates: 27°72'N, 103°92'E, altitude: 1480 m asl., on the fallen angiosperm branch, leg. C.L. Zhao, 3 Jul 2023, CLZhao 29376, GenBank: ITS = PQ166602, nLSU = PQ295861 (SWFC!).

##### Diagnosis.

It is characterized by coriaceous basidiomata with tuberculate hymenophore surface, a monomitic hyphal system with simple-septa generative hyphae, and ellipsoid to broadly ellipsoid basidiospores.

##### Etymology.

*Tuberculatum* (Lat.): refers to the species having the tuberculate basidiomata.

##### Description.

***Basidiomata*.** Annual, resupinate, closely adnate, coriaceous, without odor or taste when fresh, up to 10 cm long, 3 cm wide, and 400 μm thick. Hymenophore tuberculate, white when fresh, white to cream upon drying. Sterile margin narrow, white to cream, up to 1 mm.

**Figure 5.**
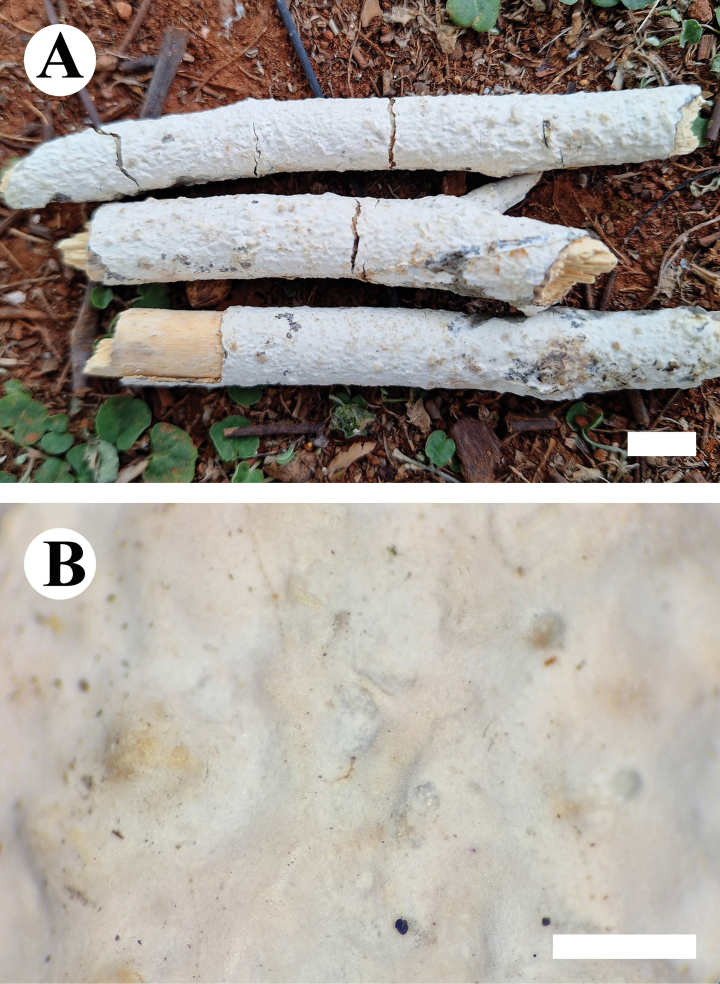
Basidiomata of *Conferticiumtuberculatum* (holotype CLZhao 29376). Scale bars: 1 cm (**A**); 1 mm (**B**).

***Hyphal system*.** Monomitic; generative hyphae simple-septate, colorless, thin-walled, smooth, rarely branched, interwoven, 3–3.5 µm in diameter, IKI+, CB–; tissues unchanged in KOH.

***Hymenium*.** Gloeocystidia of two types: (1) fusiform, often with an apical appendix, flexuous, colorless, thin-walled, smooth, 34–46 × 7–9 µm; (2) clavate, colorless, thin-walled, smooth, 36–39 × 7–8 µm. Basidia subcylindrical to subclavate, slightly flexuous, with a basal simple septum and four sterigmata, 33.5–43 × 8–10 µm; basidioles numerous, in shape similar to basidia but smaller.

**Figure 6.**
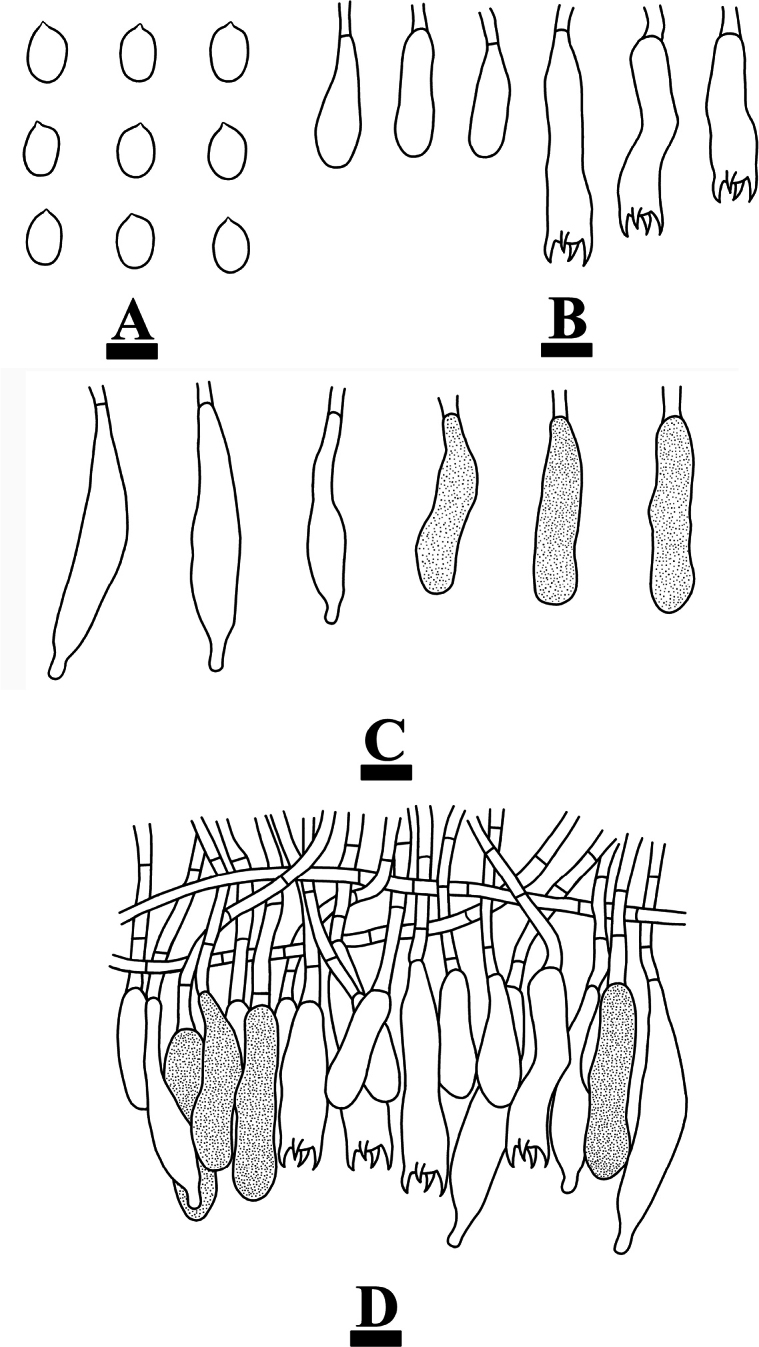
Microscopic structures of *Conferticiumtuberculatum* (holotype CLZhao 29376): basidiospores (**A**); basidia and basidioles (**B**); gloeocystidia (**C**); a section of the hymenium (**D**). Scale bars: 10 µm (**A–D**).

***Spores*.** Basidiospores ellipsoid to broadly ellipsoid, colorless, thin-walled, smooth, IKI+, CB–, (8–)8.5–11 × (5.5–)6–7.5 µm, L = 9.69 µm, W = 6.66 µm, Q = 1.46–1.58 (n = 60/2).

##### Additional specimen examined (paratype).

China. • Yunnan Province: Zhaotong, Daguan County, Huanglianhe Scenic Spot, GPS coordinates: 27°72'N, 103°92'E, altitude: 1480 m asl., on the fallen angiosperm branch, leg. C.L. Zhao, 3 Jul 2023, CLZhao 29390, GenBank: ITS = PQ166603, nLSU = PQ295862 (SWFC!).

#### ﻿ Gloeocystidiellum cremeum

Taxon classificationFungiRussulalesStereaceae

L. Wang & C.L. Zhao sp. nov.

AF8C0745-940E-508E-A32F-723AF965E878

855873

Figs 7 , 8

##### Holotype.

China. • Yunnan Province: Zhaotong, Wumengshan National Nature Reserve, GPS coordinates: 27°77'N, 104°25'E, altitude: 1900 m asl., on the fallen angiosperm branch, leg. C.L. Zhao, 20 Sep 2023, CLZhao 33623, GenBank: ITS = PQ287847, nLSU = PQ295864 (SWFC!).

##### Diagnosis.

It is characterized by cream membranaceous basidiomata, a monomitic hyphal system with clamped generative hyphae, thick-walled, subcylindrical to obclavate gloeocystidia, and ellipsoid to subglobose basidiospores.

**Figure 7.**
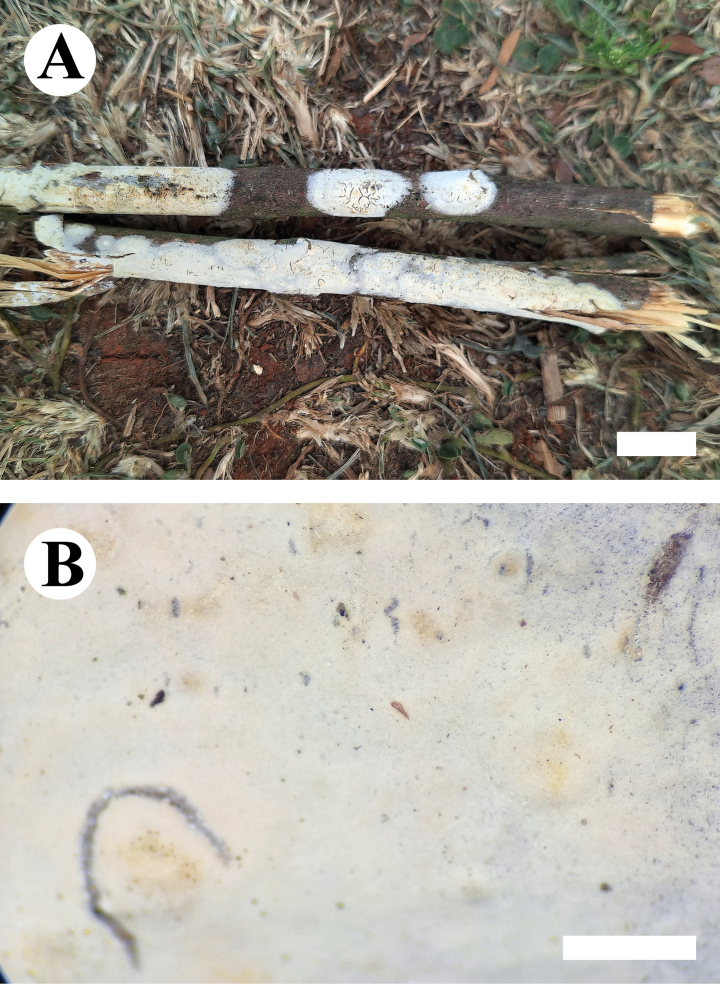
Basidiomata of *Gloeocystidiellumcremeum* (holotype CLZhao 33623). Scale bars: 1 cm (**A**); 1 mm (**B**).

##### Etymology.

*Cremeum* (Lat.): refers to the species having a cream color of the hymenial surface.

##### Description.

***Basidiomata*.** Annual, resupinate, closely adnate, membranaceous, without odor or taste when fresh, up to 8.5 cm long, 2 cm wide, and 300 μm thick. Hymenophore smooth, white when fresh, white to cream upon drying. Sterile margin cream, up to 1 mm.

**Figure 8.**
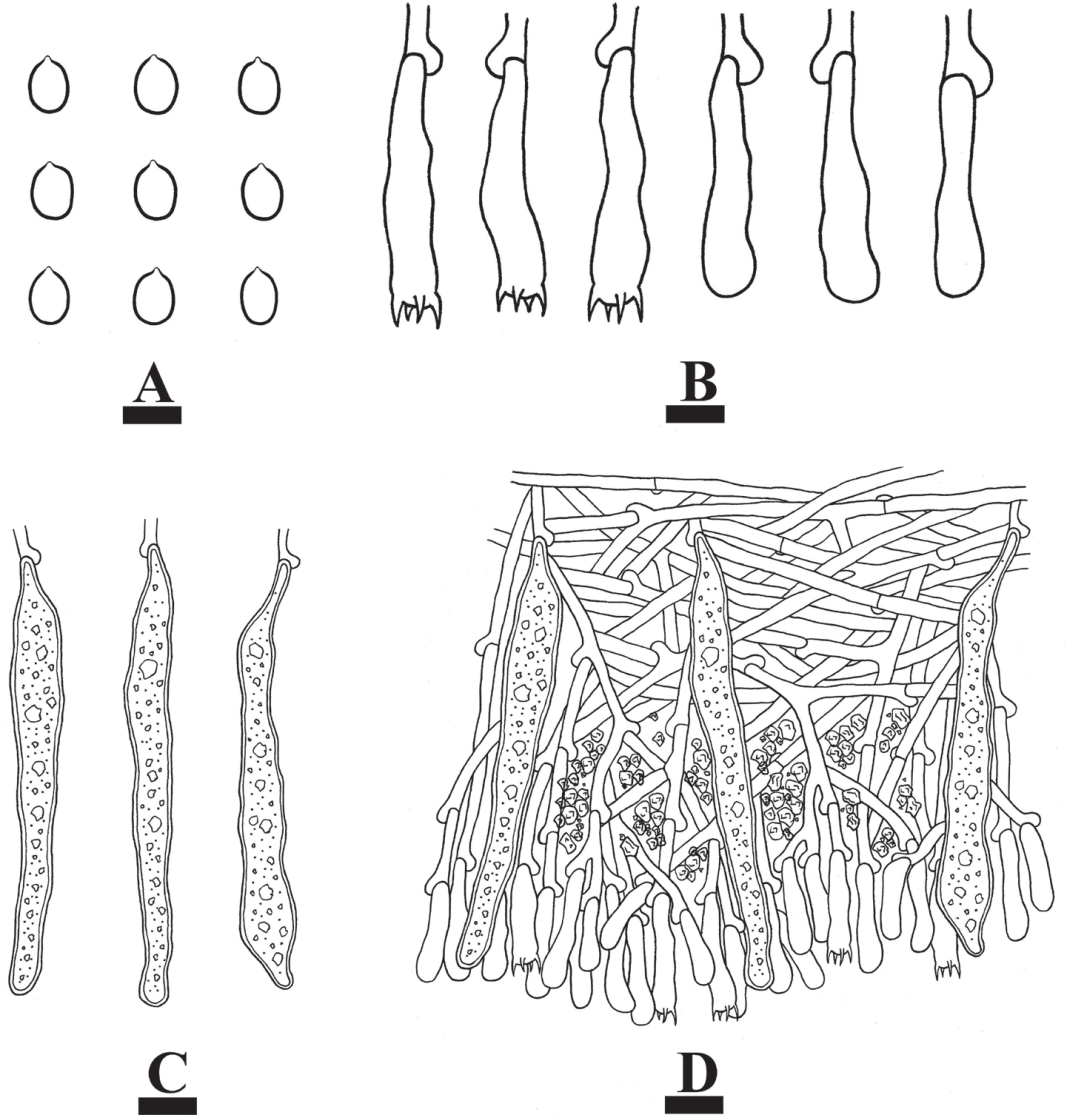
Microscopic structures of *Gloeocystidiellumcremeum* (holotype CLZhao 33623): basidiospores (**A**); basidia and basidioles (**B**); gloeocystidia (**C**); a section of the hymenium (**D**). Scale bars: 5 µm (**A, B**); 10 µm (**C, D**).

***Hyphal system*.** Monomitic; generative hyphae with clamp connections, colorless, thin-walled, smooth, branched, interwoven, 2–3 µm in diameter, IKI–, CB–; tissues unchanged in KOH.

***Hymenium*.** Gloeocystidia numerous, variable in size and shape, subcylindrical to obclavate, colorless, slightly thick-walled, smooth, mostly 70–77 × 7.5–10.5 µm. Basidia subcylindrical to subclavate, slightly flexuous, with a basal clamp connection and four sterigmata, 19–24 × 3–4.5 µm; basidioles numerous, in shape similar to basidia but smaller.

***Spores*.** Basidiospores ellipsoid to subglobose, colorless, thin-walled, smooth, IKI+, CB–, (3.5–)4–5 × (2–)2.5–3.5 µm, L = 4.41 µm, W = 2.97 µm, Q = 1.39–1.51 (n = 90/3).

##### Additional specimens examined (paratypes).

China. • Yunnan Province: Zhaotong, Weixin County, Tianxing National Forest Park, GPS coordinates: 28°05'N, 105°09'E, altitude: 900 m asl., on the fallen angiosperm branch, leg. C.L. Zhao, 5 Jul 2023, CLZhao 29477, GenBank: ITS = PQ287846, nLSU = PQ295863; • Zhaotong, Wumengshan National Nature Reserve, GPS coordinates: 27°77'N, 104°25'E, altitude: 1900 m asl., on the fallen angiosperm branch, leg. C.L. Zhao, 20 Sep 2023, CLZhao 33690, GenBank: ITS = PQ287848 (SWFC!).

#### ﻿ Gloeocystidiellum fissuratum

Taxon classificationFungiRussulalesStereaceae

L. Wang & C.L. Zhao sp. nov.

84BA4B26-374C-556C-A2D7-FB011E65BAAA

855874

Figs 9 , 10

##### Holotype.

China. • Yunnan Province: Zhaotong, Wumengshan National Nature Reserve, GPS coordinates: 27°77'N, 104°25'E, altitude: 1900 m asl., on the fallen angiosperm branch, leg. C.L. Zhao, 28 Aug 2023, CLZhao 32498, GenBank: ITS = PQ287851 (SWFC!).

##### Diagnosis.

It is characterized by white to cinnamon-buff, membranaceous basidiomata with grandinioid and cracking hymenophore surfaces, a monomitic hyphal system with clamped generative hyphae, numerous, variable in size and shape gloeocystidia, and subglobose basidiospores.

##### Etymology.

*Fissuratum* (Lat.): refers to the species having a cracking hymenial surface.

##### Description.

***Basidiomata*.** Annual, resupinate, closely adnate, membranaceous, without odor or taste when fresh, up to 8.5 cm long, 2 cm wide, and 300 μm thick. Hymenophore grandinioid, cracking, white to cinnamon-buff when fresh, cinnamon-buff upon drying. Sterile margin cream, up to 2 mm.

**Figure 9.**
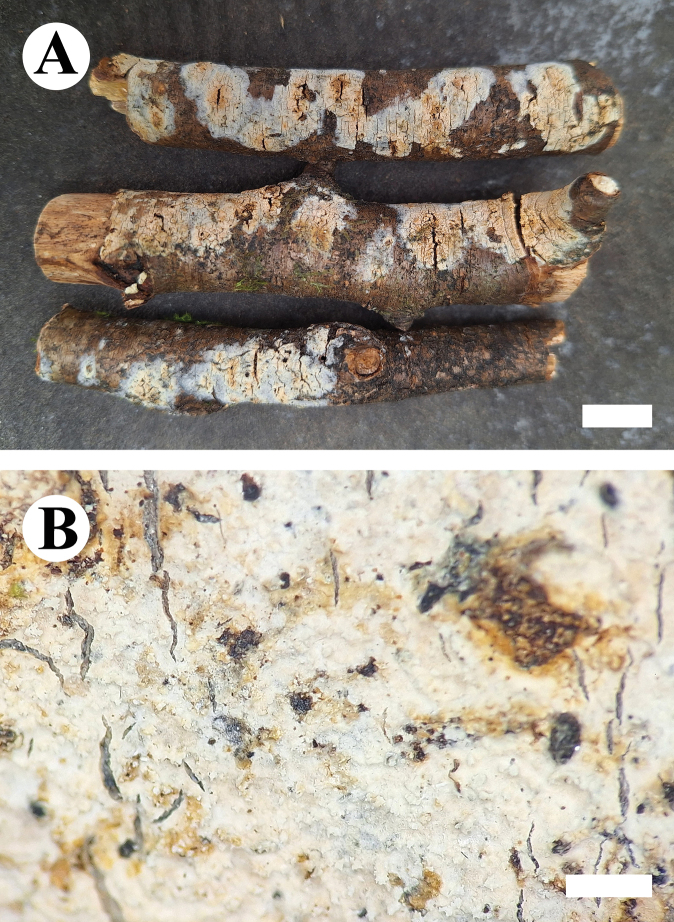
Basidiomata of *Gloeocystidiellumfissuratum* (holotype CLZhao 32498). Scale bars: 1 cm (**A**); 1 mm (**B**).

***Hyphal system*.** Monomitic; generative hyphae with clamp connections, colorless, thin-walled, smooth, branched, interwoven, 2–3 µm in diameter, IKI–, CB–; tissues unchanged in KOH.

***Hymenium*.** Gloeocystidia numerous, variable in size and shape, subclavate to obclavate, colorless, thin-walled, smooth, mostly 57–88 × 9–10 µm. Basidia subcylindrical to subclavate, slightly flexuous, with a basal clamp connection and four sterigmata, 13–16 × 4–5 µm; basidioles numerous, in shape similar to basidia but smaller.

**Figure 10.**
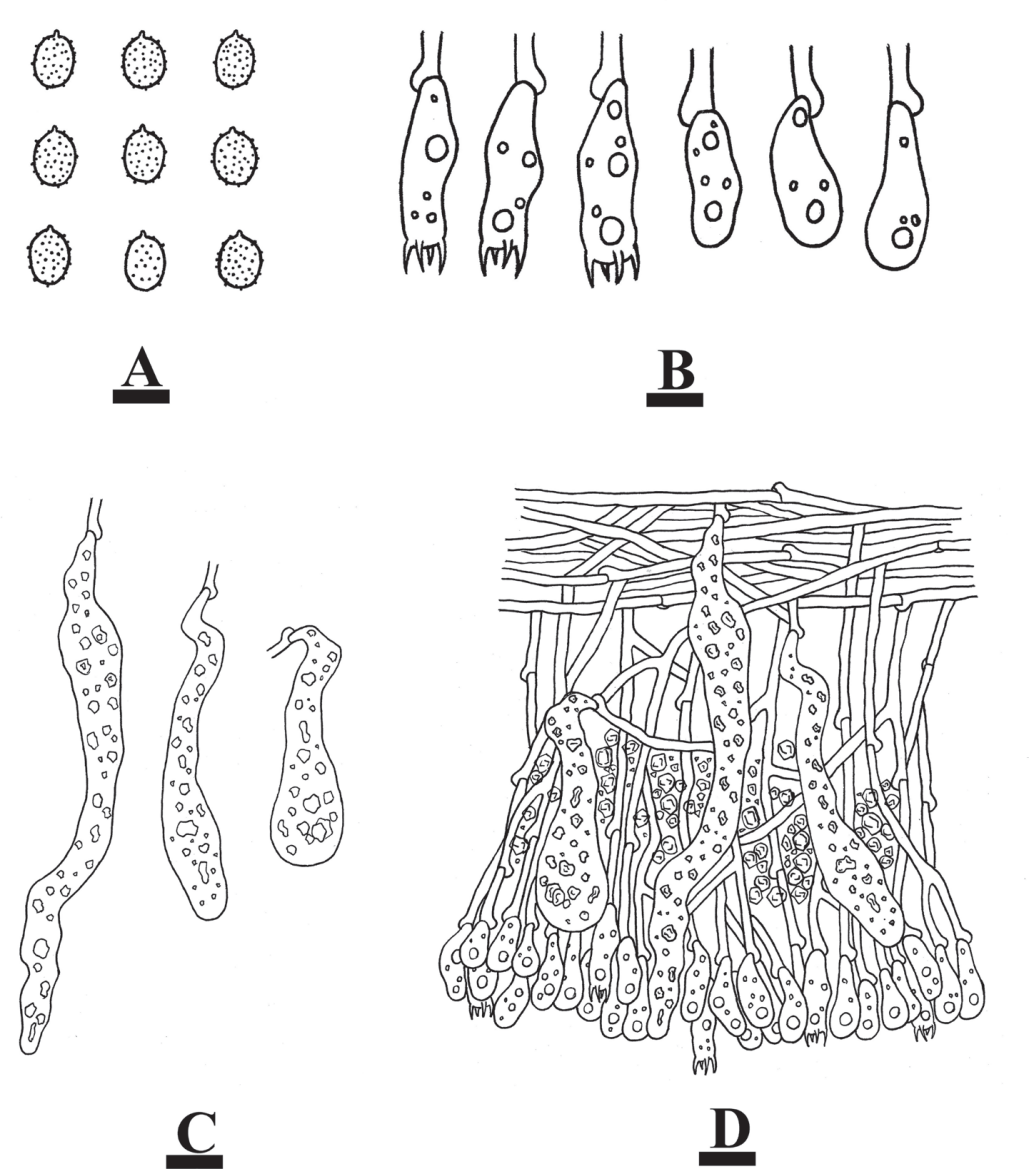
Microscopic structures of *Gloeocystidiellumfissuratum* (holotype CLZhao 32498): basidiospores (**A**); basidia and basidioles (**B**); gloeocystidia (**C**); a section of the hymenium (**D**). Scale bars: 5 µm (**A, B**); 10 µm (**C, D**).

***Spores*.** Basidiospores subglobose, colorless, thin-walled, verrucose, IKI+, CB–, (3–)3.5–4.5(–5) × 2.5–3.5(–4) µm, L = 4.04 µm, W = 3.06 µm, Q = 1.26–1.32 (n = 90/3).

##### Additional specimens examined (paratypes).

China. • Yunnan Province: Zhaotong, Wumengshan National Nature Reserve, GPS coordinates: 27°77'N, 104°25'E, altitude: 1900 m asl., on angiosperm stump, leg. C.L. Zhao, 28 Aug 2023, CLZhao 32247, GenBank: ITS = PQ287849; nLSU = PQ295865; on the fallen branch of *Picea*, leg. C.L. Zhao, 28 Aug 2023, CLZhao 32303, GenBank: ITS = PQ287850, nLSU = PQ295866 (SWFC!).

#### ﻿ Peniophora albohymenia

Taxon classificationFungiRussulalesPeniophoraceae

L. Wang & C.L. Zhao sp. nov.

4E28AFD4-2B54-5957-8219-19FC13969953

855875

Figs 11 , 12

##### Holotype.

China. • Yunnan Province: Zhaotong, Fenghuangshan National Forest Park, GPS coordinates: 27°30'N, 103°70'E, altitude: 1950 m asl., on the fallen angiosperm branch, leg. C.L. Zhao, 24 Aug 2022, CLZhao 23473, GenBank: ITS = PQ066419, nLSU = PQ295867 (SWFC!).

##### Diagnosis.

It is characterized by white to pale pink, smooth membranaceous basidiomata, a monomitic hyphal system with simple-septa generative hyphae, and allantoid to cylindrical basidiospores.

##### Etymology.

*Albohymenia* (Lat.): refers to the species having white basidiomata.

##### Description.

***Basidiomata*.** Annual, resupinate, closely adnate, membranaceous, without odor or taste when fresh, up to 9 cm long, 3.5 cm wide, and 300 μm thick. Hymenophore smooth, white when fresh, white to pale pink upon drying. Sterile margin narrow, white, up to 1 mm.

**Figure 11.**
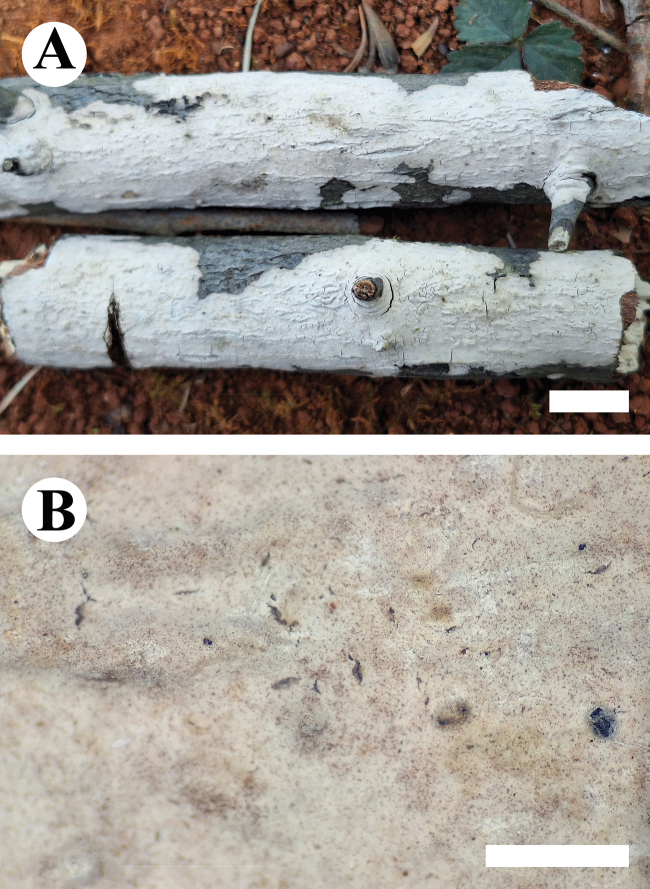
Basidiomata of *Peniophoraalbohymenia* (holotype CLZhao 23473). Scale bars: 1 cm (**A**); 1 mm (**B**).

***Hyphal system*.** Monomitic; generative hyphae with simple-septa, colorless, slightly thick-walled, smooth, rarely branched and septate, more or less parallel to substrate, 4–4.5 µm in diameter, IKI–, CB–; tissues unchanged in KOH.

***Hymenium*.** Cystidia of two types: (1) Gloeocystidia fusiform, flexuous, colorless, thin-walled, smooth, 31.5–35.5 × 6–7 µm; (2) Lamprocystidia abundant, subulate to subcylindrical, heavily encrusted with crystals in the middle and upper parts, thin-walled, colorless, embedded or projecting beyond the hymenium, with a basal simple-septum, 31–42 × 10–13.5 µm. Basidia subcylindrical to subclavate, slightly flexuous, with a basal simple septum and four sterigmata, 23.5–26 × 4.5–6 µm; basidioles numerous, in shape similar to basidia but slightly smaller.

**Figure 12.**
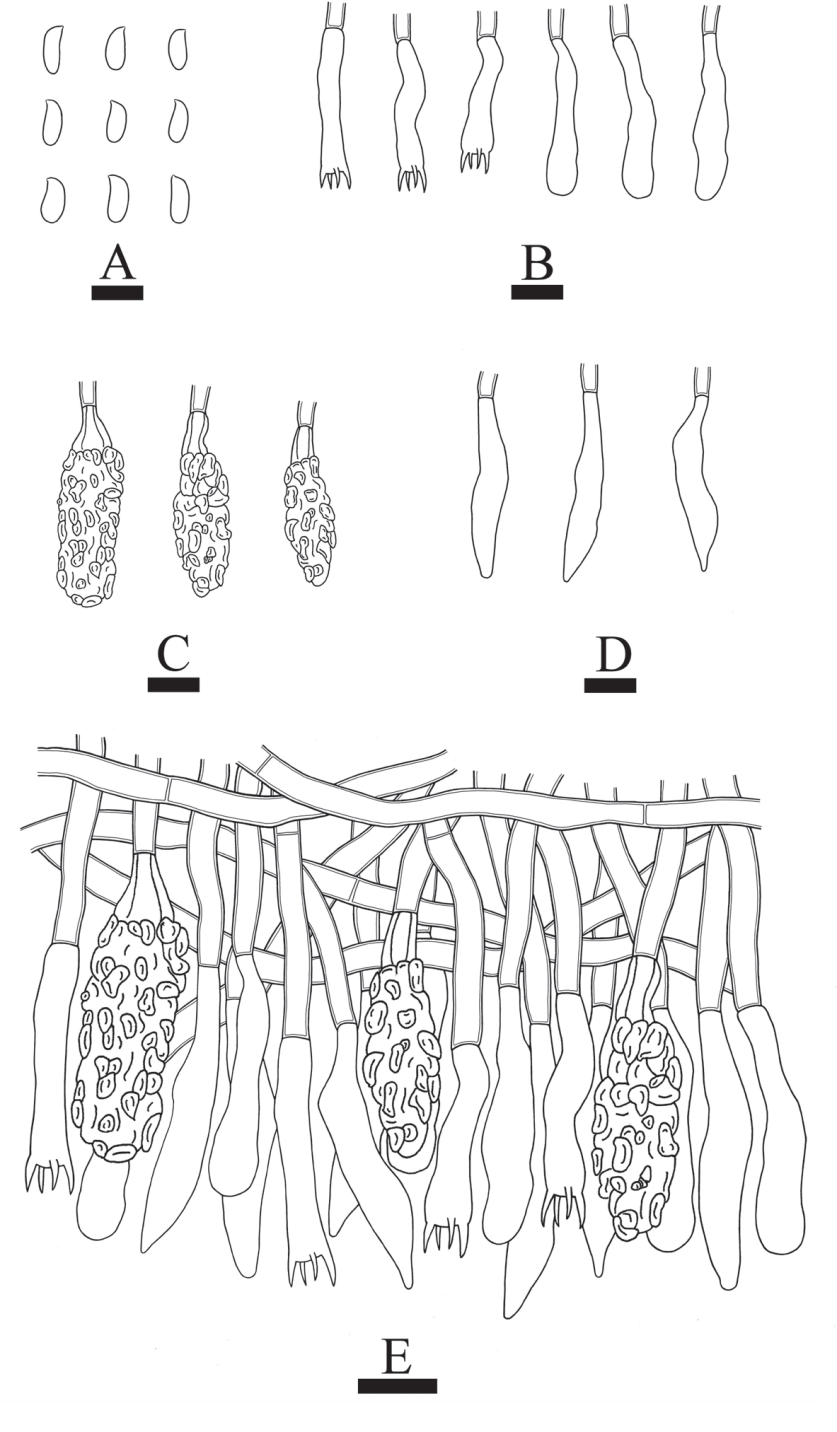
Microscopic structures of *Peniophoraalbohymenia* (holotype CLZhao 23473): basidiospores (**A**); basidia and basidioles (**B**); lamprocystidia (**C**); gloeocystidia (**D**); a section of the hymenium (**E**). Scale bars: 10 µm (**A–E**).

***Spores*.** Basidiospores allantoid to cylindrical, colorless, thin-walled, smooth, IKI–, CB–, (7–)8.5–11(–11.5) × 3–4.5(–5) µm, L = 9.65 µm, W = 3.93 µm, Q = 2.46 (n = 30/1).

#### ﻿ Peniophora hengduanensis

Taxon classificationFungiRussulalesPeniophoraceae

L. Wang & C.L. Zhao sp. nov.

6ACC77E5-A3BB-5859-A7E3-0FB22945C9D4

855876

Figs 13 , 14

##### Holotype.

China. • Yunnan Province: Diqing, Weixi County, Zhonglu Town, GPS coordinates: 27°16'N, 99°15'E, altitude: 2250 m asl., on the fallen angiosperm branch, leg. C.L. Zhao, 14 Oct 2022, CLZhao 34697, GenBank: ITS = PQ066422, nLSU = PQ295870 (SWFC!).

##### Diagnosis.

It is characterized by pink to vinaceous, smooth membranaceous basidiomata, a monomitic hyphal system with simple-septa, and allantoid to subcylindrical basidiospores.

##### Etymology.

*Hengduanensis* (Lat.): refers to the locality (Hengduan Mountains) of the type specimen.

##### Description.

***Basidiomata*.** Annual, resupinate, closely adnate, membranaceous, without odor or taste when fresh, up to 7.5 cm long, 4 cm wide, and 400 μm thick. Hymenophore smooth, pale pink when fresh, pink to vinaceous upon drying. Sterile margin narrow, white to vinaceous, up to 1 mm.

**Figure 13.**
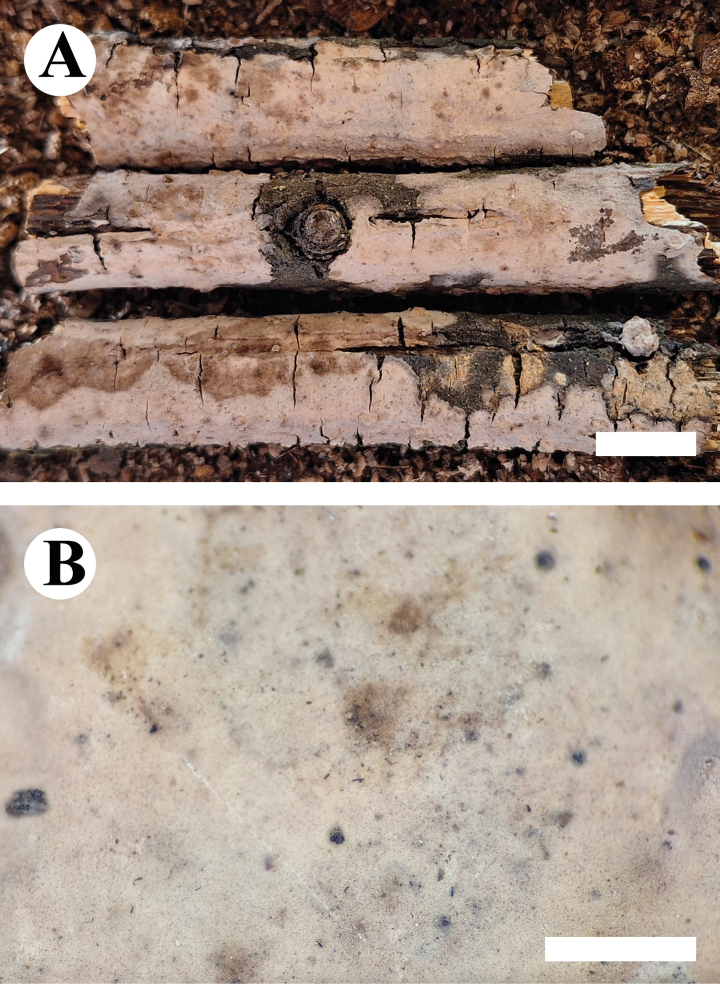
Basidiomata of *Peniophorahengduanensis* (holotype CLZhao 34697). Scale bars: 1 cm (**A**); 1 mm (**B**).

***Hyphal system*.** Monomitic; generative hyphae with simple-septa, colorless, thin-walled, smooth, rarely branched, rarely septate, more or less parallel to substrate, 3.5–4.5 µm in diameter, IKI–, CB–; tissues unchanged in KOH.

***Hymenium*.** Cystidia of two types: (1) Gloeocystidia obclavate, colorless, slightly thick-walled, smooth, 50.5–66 × 11–14.5 µm; (2) Lamprocystidia abundant, subulate to subcylindrical, heavily encrusted with crystals in the middle and upper parts, thin-walled, colorless, embedded or projecting beyond the hymenium, with a basal simple septum, 21.5–25 × 9.5–11 µm. Basidia subcylindrical to subclavate, slightly flexuous, with a basal simple septum and four sterigmata, 20–32.5 × 4.5–6 µm; basidioles numerous, in shape similar to basidia but slightly smaller.

**Figure 14.**
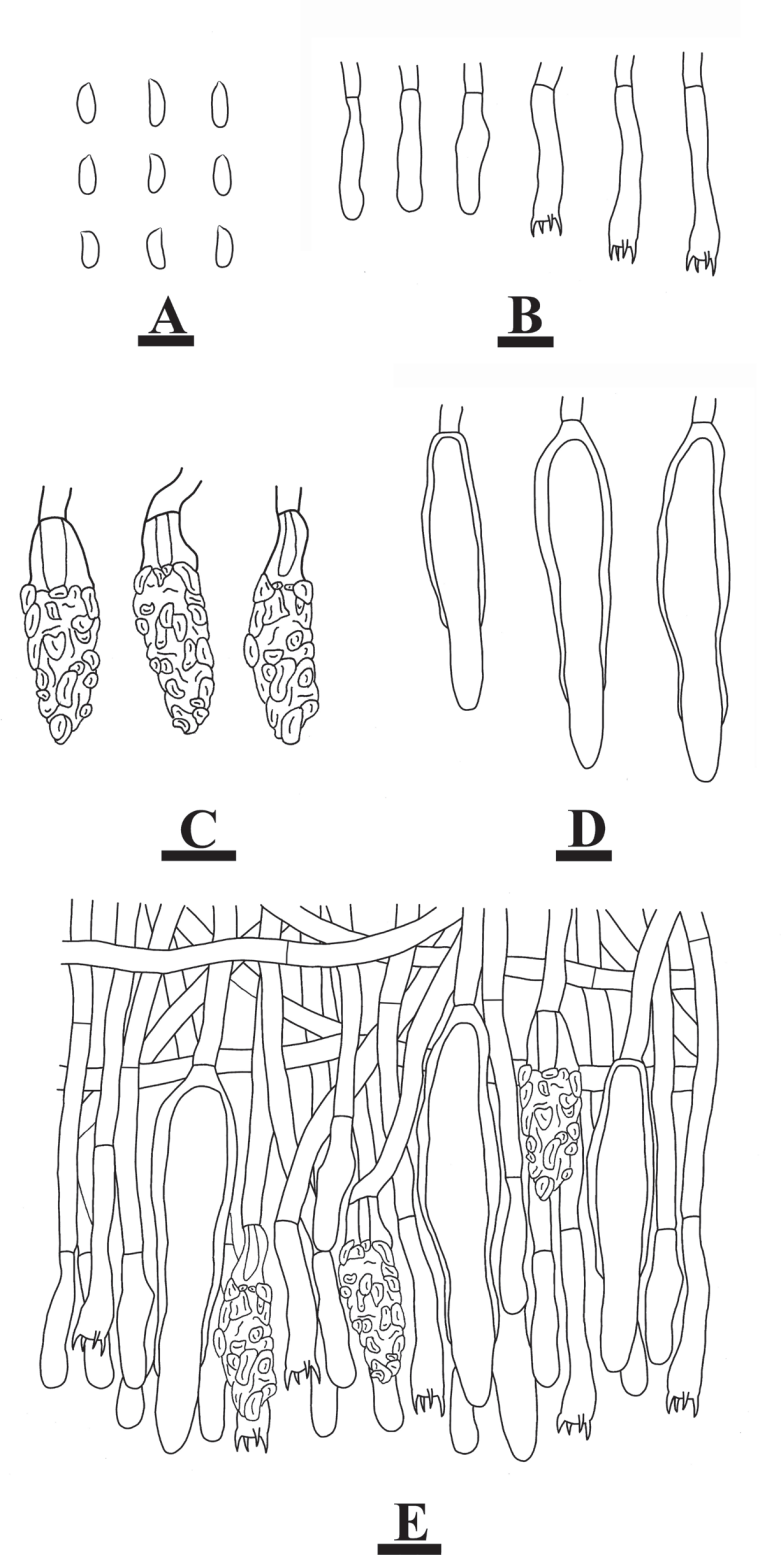
Microscopic structures of *Peniophorahengduanensis* (holotype CLZhao 34697): basidiospores (**A**); basidia and basidioles (**B**); lamprocystidia (**C**); gloeocystidia (**D**); a section of the hymenium (**E**). Scale bars: 10 µm (**A–E**).

***Spores*.** Basidiospores allantoid to subcylindrical, colorless, thin-walled, smooth, IKI–, CB–, (6–)6.5–8.5(–9) × 2.5–3.5(–4) µm, L = 7.35 µm, W = 3.15 µm, Q = 2.33 (n = 30/1).

#### ﻿ Peniophora punctata

Taxon classificationFungiRussulalesPeniophoraceae

L. Wang & C.L. Zhao sp. nov.

A7AE0472-AB0C-5133-8CB4-D69893578535

855877

Figs 15 , 16

##### Holotype.

China. • Yunnan Province: Zhaotong, Xiaocaoba, Wumengshan National Nature Reserve, GPS coordinates: 27°77'N, 104°25'E, altitude: 1900 m asl., on the fallen angiosperm branch, leg. C.L. Zhao, 21 Sep 2023, CLZhao 33769, GenBank: ITS = PQ066418 (SWFC!).

##### Diagnosis.

It is characterized by pink to slightly purple, cushion-shaped, smooth membranaceous basidiomata, a monomitic hyphal system with simple-septa, thick-walled generative hyphae, and allantoid basidiospores.

##### Etymology.

*Punctata* (Lat.): refers to the species having cushion-shaped basidiomata.

##### Description.

***Basidiomata*.** Annual, resupinate, closely adnate, cushion-shaped, membranaceous, without odor or taste when fresh, up to 3 cm long, 1.5 cm wide, and 300 μm thick. Hymenophore smooth, pink to slightly purple when fresh, purple upon drying. Sterile margin narrow, white to vinaceous, up to 1 mm.

**Figure 15.**
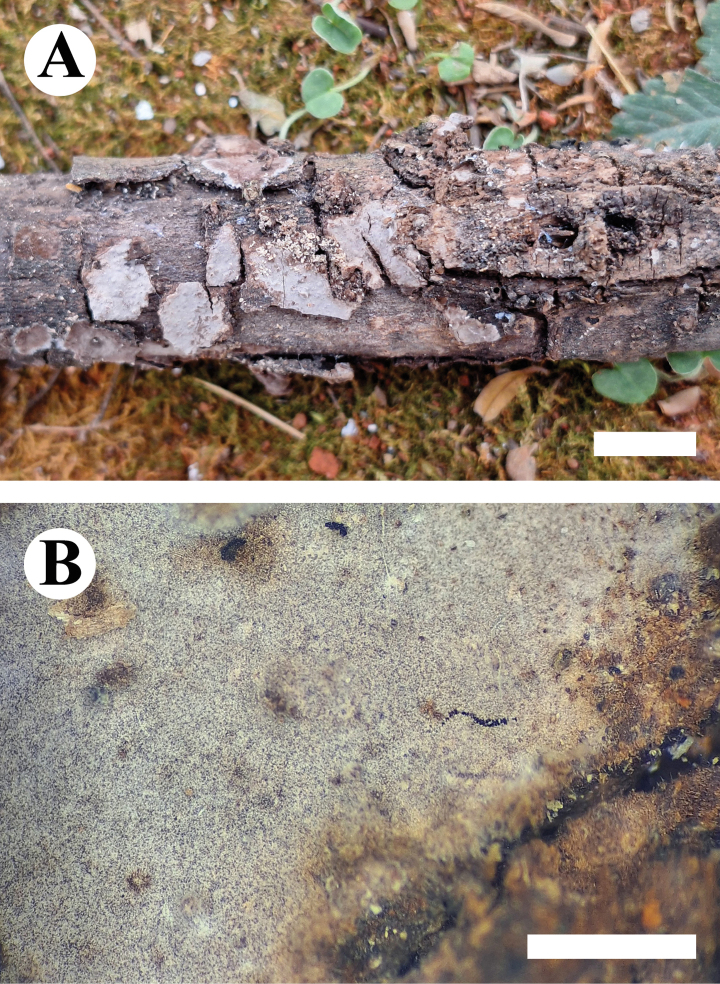
Basidiomata of *Peniophorapunctata* (holotype CLZhao 33769). Scale bars: 1 cm (**A**); 1 mm (**B**).

***Hyphal system*.** Monomitic; generative hyphae with simple-septa, colorless, slightly thick-walled, smooth, rarely branched, rarely septate, more or less parallel to substrate, 2.5–3.5 µm in diameter, IKI–, CB–; tissues unchanged in KOH.

***Hymenium*.** Cystidia of two types: (1) Gloeocystidia fusiform or subclavate, slightly flexuous, colorless, slightly thick-walled, smooth, 31.5–51.5 × 6–8 µm; (2) Lamprocystidia abundant, subulate to subcylindrical, heavily encrusted with crystals in the middle and upper parts, thin-walled, colorless, embedded or projecting beyond the hymenium, with a basal simple septum, 36–39 × 8.5–12.5 µm. Basidia subcylindrical to subclavate, slightly flexuous, with a basal simple septum and four sterigmata, 18–21.5 × 3–4.5 µm; basidioles numerous, in shape similar to basidia but slightly smaller.

**Figure 16.**
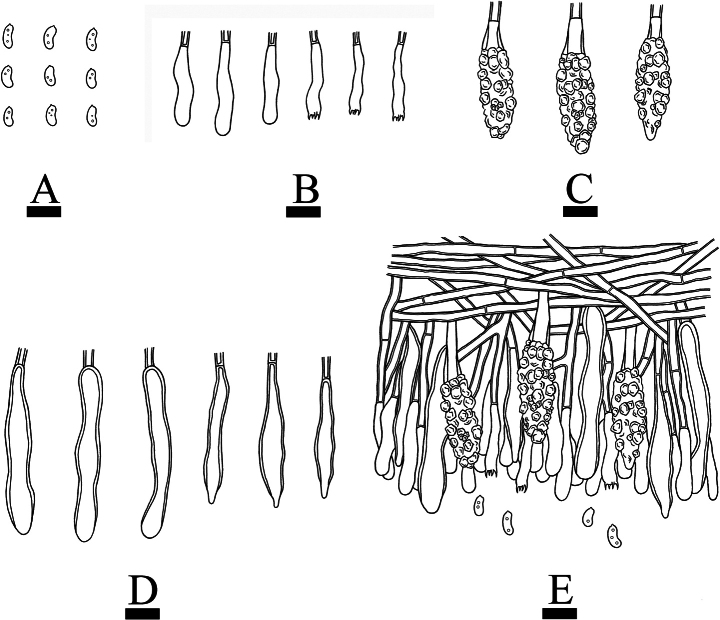
Microscopic structures of *Peniophorapunctata* (holotype CLZhao 33769): basidiospores (**A**); basidia and basidioles (**B**); lamprocystidia (**C**); gloeocystidia (**D**); a section of the hymenium (**E**). Scale bars: 10 µm (**A–E**).

***Spores*.** Basidiospores allantoid, colorless, thin-walled, smooth, IKI–, CB–, 5–7(–7.5) × 1.5–2.5 µm, L = 6.08 µm, W = 2.22 µm, Q = 2.73 (n = 30/1).

## ﻿Discussion

A large number of studies were focused on the taxonomy and phylogeny of Russulales taxa in the last ten years (He et al. 2019; Wijayawardene et al. 2022; Wu et al. 2020; Zou et al. 2022; Li et al. 2023; Deng et al. 2024b; Dong et al. 2024; Liu et al. 2024; Zhou et al. 2024). Both families, Peniophoraceae and Stereaceae, are well-supported large groups in Russulales, and most species have resupinate and effused-reflexed basidiomata growing on fallen twigs, branches, or trunks of woody plants or bamboos (Larsson and Larsson 2003; Miller et al. 2006; Zhou and Dai 2013; De Crop et al. 2016; He et al. 2024). Molecular analyses have elucidated the evolutionary relationships, in which the findings demonstrate significant morphological changes occurring at both the levels of family and genus (Larsson and Larsson 2003; Miller et al. 2006; Zhao et al. 2017; He et al. 2019; Wu et al. 2020). Elucidating morphological differences for its overall appearance, structure, colors, spore characters, and hyphal structure, as well as its habitat, this distinction holds significant importance for making phylogenetic and systematic conclusions (Miller et al. 2006; Dong et al. 2024; He et al. 2024).

Phylogenetically, the multiple genes with ITS+nLSU analysis showed that the six new species grouped within the order Russulales, in which *Conferticiumtuberculatum*, *Gloeocystidiellumcremeum*, and *G.fissuratum* grouped into the family Stereaceae. *Conferticiumtuberculatum* is separated from closely related species *C. ravum*, which can be delimited from *C. tuberculatum* by its smooth, yellowish to isabelline hymenophore, shorter basidia (20–30 × 4–6 µm), and ellipsoid to ovoid, verrucose basidiospores (6–7 × 4–4.5 µm; Bernicchia and Gorjón 2010). Based on the ITS+nLSU sequence data (Fig. 2), *Gloeocystidiellumcremeum* is grouped with *G.fissuratum* L. Wang & C.L. Zhao and *G.yunnanense* Y.L. Zhao & C.L. Zhao in the *Gloeocystidiellum* clade. However, *G.fissuratum* differs from *G.cremeum* by its white to cinnamon-buff, grandinioid, and cracking hymenophore and smaller basidia (13–16 × 4–5 µm); *G.yunnanense* is distinguished from *G.cremeum* by its cream, ceraceous, and grandinioid hymenophore, smaller basidia (12.5–14.5 × 3.5–4.5 µm), and slightly thick-walled, aculeate, ellipsoid basidiospores (Zhao and Zhao 2023). *Gloeocystidiellumfissuratum* is distinguished from *G.yunnanense* by cream, ceraceous, and grandinioid hymenophore, smaller basidia (12.5–14.5 × 3.5–4.5 µm), and slightly thick-walled, aculeate, ellipsoid basidiospores (Zhao and Zhao 2023).

Based on the ITS+nLSU analysis, three new taxa, *Peniophoraalbohymenia*, *P.hengduanensis*, and *P.punctata*, were grouped into the family Peniophoraceae. As inferred from the sequence data (Fig. 4), *Peniophoraalbohymenia* is a sister of *P.reidii* Boidin & Lanq. However, *P.reidii* is distinguished from *P.albohymenia* by its pinkish gray to gray basidiomata and longer gloeocystidia (25–75 × 5.5–7.5 µm; Boidin and Lanquetin 1983). Based on the ITS+nLSU sequence data (Fig. 4), *Peniophorahengduanensis* formed a sister group with *P.crassitunicata* Boidin, Lanq. However, *P.crassitunicata* is distinguished from *P.hengduanensis* by its pinkish gray to grayish violaceous basidiomata, thick-walled generative hyphae, and bigger, very thick-walled (2–3 µm) gloeocystidia (60–115 × 8–15 µm) (Boidin and Lanquetin 1983). *Peniophorapunctata* is a closely related species, viz., *P.borbonica* Boidin, Lanq, and *P.laxitexta* C.E. Gómez. However, *P.borbonica* is delimited from *P.punctata* by its smooth, purplish gray or gray hymenophore, longer gloeocystidia (30–60 × 7–12 µm), and suballantoid to cylindrical, bigger basidiospores (8–10.5 × 2.7–3.5 µm; Boidin and Gilles 2000); *P.laxitexta* can be distinguished from *P.punctata* by its longer basidia (27–35 × 4.5–6 µm) and bigger lamprocystidia (30–60 × 10–22 µm; Gómez and Loewenbaum 1976).

Morphologically, *Conferticiumochraceum* (Fr.) Hallenb. is similar to *C. tuberculatum* by having smooth ceraceous basidiomata. However, *C. ochraceum* differs in its coriaceous basidiomata with pale yellowish hymenophore surface and subcylindrical to subovate basidiospores (4–6.5 × 2.5–3.5 µm; Bernicchia and Gorjón 2010). *Gloeocystidiellumclavuligerum* (Höhn. and Litsch.) Nakasone and *G.porosum* (Berk. and M.A. Curtis) Donk are similar to *G.cremeum* by both having ellipsoid to subglobose basidiospores. However, *G.clavuligerum* differs in its gloeocystidia with a more or less constricted to moniliform apex (50–80 × 8–12 µm; Bernicchia and Gorjón 2010); *G.porosum* differs in its bigger gloeocystidia (80–200 × 8–15 µm, Bernicchia and Gorjón 2010). *Gloeocystidiellumclavuligerum* and *G.porosum* are similar to *G.fissuratum* by both having verrucose, thin-walled basidiospores. However, *G.clavuligerum* differs in its longer basidia (25–30 × 4–5 µm; Bernicchia and Gorjón 2010); *G.porosum* differs in its bigger gloeocystidia (80–200 × 8–15 µm; Bernicchia and Gorjón 2010).

Morphologically, *Peniophoraroseoalba* L. Zou & C.L. Zhao and *P.shenghuae* Y.L. Xu, Yan Tian & S.H. He are similar to *P.albohymenia* by both having fusiform, flexuous gloeocystidia. However, *P.roseoalba* differs in its ellipsoid basidiospores (4–6.5 × 3–5 µm; Zou et al. 2022); *P.shenghuae* differs in its coriaceous basidiomata with a brownish-orange hymenophore surface (Xu et al. 2023). *Peniophoravietnamensis* Y.L. Xu, Y. Tian & S.H. He, and *P.pini* (Schleich. ex DC.) Boidin are similar to *P.hengduanensis* by having subcylindrical basidia. However, *P.vietnamensis* differs in its oblong cylindrical basidiospores (14–17 × 4–6 µm; Xu et al. 2023); *P.pini* differs in its clamped generative hyphae and longer lamprocystidia (25–40 × 5–8 µm; Bernicchia and Gorjón 2010). *Peniophoracrassitunicata* Boidin, Lanq. & Gilles, and *P.pithya* (Pers.) J. Erikss. are similar to *P.punctata* by having allantoid basidiospores. However, *P.crassitunicata* differs in its clamped generative hyphae and longer subcylindrical basidia (25–40 × 4.5–6 µm; Andreasen and Hallenberg 2009); *P.pithya* differs in its clamped generative hyphae and longer lamprocystidia (30–70 × 8–15 µm; Bernicchia and Gorjón 2010).

Ecological functions performed by members of the order Russulales include mycorrhizal symbiosis and wood decay, which play an important role in nutrient cycling and decomposition within forest ecosystems (Cui et al. 2019; Wu et al. 2019, 2022; Liu et al. 2023; Dai et al. 2021; Deng et al. 2024b; Hyde et al. 2024; Wang et al. 2024a, b; Zhao et al. 2024). Although in the Basidiomycota, there has been a clear evolutionary trend in the development of different types of basidiomata, the taxonomy and phylogeny of some taxa in the order Russulales are still unresolved (Larsson and Larsson 2003; Miller et al. 2006; He et al. 2019, 2024; Wijayawardene et al. 2022, 2024; Wu et al. 2020; Bhunjun et al. 2024; Dong et al. 2024; Liu et al. 2024). In the present study, these data are also crucial as a supplement to the global knowledge of wood-inhabiting of the order Russulales.

## Supplementary Material

XML Treatment for Conferticium tuberculatum

XML Treatment for Gloeocystidiellum cremeum

XML Treatment for Gloeocystidiellum fissuratum

XML Treatment for Peniophora albohymenia

XML Treatment for Peniophora hengduanensis

XML Treatment for Peniophora punctata

## ﻿Additional information

### Author ORCIDs

Lu Wang https://orcid.org/0009-0004-6274-5953

Yonggao Zhu https://orcid.org/0009-0008-5341-3798

Yonggao Zhu https://orcid.org/0009-0005-2478-0624

Sana Jabeen https://orcid.org/0000-0001-8839-7716

Changlin Zhao https://orcid.org/0000-0002-8668-1075

### Data availability

All of the data that support the findings of this study are available in the main text.
